# Development of a risk model to predict prognosis in breast cancer based on cGAS-STING-related genes

**DOI:** 10.3389/fgene.2023.1121018

**Published:** 2023-03-27

**Authors:** Chen Chen, Junxiao Wang, Chao Dong, David Lim, Zhihui Feng

**Affiliations:** ^1^ Department of Occupational Health and Occupational Medicine, School of Public Health, Cheeloo College of Medicine, Shandong University, Jinan, China; ^2^ Translational Health Research Institute, School of Health Sciences, Western Sydney University, Campbelltown, NSW, Australia; ^3^ College of Medicine and Public Health, Flinders University, Adelaide, SA, Australia

**Keywords:** breast cancer, cGAS-STING pathway, risk score model, prognosis, immunothearpy

## Abstract

**Background:** Breast cancer (BRCA) is regarded as a lethal and aggressive cancer with increasing morbidity and mortality worldwide. cGAS-STING signaling regulates the crosstalk between tumor cells and immune cells in the tumor microenvironment (TME), emerging as an important DNA-damage mechanism. However, cGAS-STING-related genes (CSRGs) have rarely been investigated for their prognostic value in breast cancer patients.

**Methods:** Our study aimed to construct a risk model to predict the survival and prognosis of breast cancer patients. We obtained 1087 breast cancer samples and 179 normal breast tissue samples from the Cancer Genome Atlas (TCGA) and Genotype-Tissue Expression (GTEX) database, 35 immune-related differentially expression genes (DEGs) from cGAS-STING-related genes were systematically assessed. The Cox regression was applied for further selection, and 11 prognostic-related DEGs were used to develop a machine learning-based risk assessment and prognostic model.

**Results:** We successfully developed a risk model to predict the prognostic value of breast cancer patients and its performance acquired effective validation. The results derived from Kaplan-Meier analysis revealed that the low-risk score patients had better overall survival (OS). The nomogram that integrated the risk score and clinical information was established and had good validity in predicting the overall survival of breast cancer patients. Significant correlations were observed between the risk score and tumor-infiltrating immune cells, immune checkpoints and the response to immunotherapy. The cGAS-STING-related genes risk score was also relevant to a series of clinic prognostic indicators such as tumor staging, molecular subtype, tumor recurrence, and drug therapeutic sensibility in breast cancer patients.

**Conclusion:** cGAS-STING-related genes risk model provides a new credible risk stratification method to improve the clinical prognostic assessment for breast cancer.

## Introduction

Breast cancer (BRCA) is a life-threatening disease in females, it strikes >1.6 million women worldwide and accounts for about 23% of all malignancy death (Akram et al., 2017; Harbeck and Gnant, 2017). Advances in early detection and treatment have positively impacted breast cancer mortality (Akram et al., 2017). However, the application of conventional therapeutic strategies, such as surgery, radiotherapy or chemotherapy, paradoxically may induce tumor cell metastasis and drug resistance, thus affecting the prognosis and overall survival (OS) of patients (McDonald et al., 2016; Deng et al., 2017; Ben-Dror et al., 2022). At present, prognosis-related mechanisms of BRCA remain ambiguous. Therefore, an intuitive and effective prognosis tool may be needed.

In recent times, immunotherapy has evolved as a promising anti-tumor strategy and has acquired enormous breakthroughs in treating malignant tumors (Irvine and Dane, 2020; O'Donnell et al., 2019). Accumulating data have indicated that immune response-related genes have potential to predict therapeutic response and long-term survival in patients with breast cancer (Denkert et al., 2010; Savas et al., 2016; Emens, 2018). The process of tumor immunotherapy predominantly relies on the recruitment of tumor-specific effector T cells through the antigen-bound Class I and II major histocompatibility complex (MHC-I and MHC-II) expressed on antigen-presenting cells (APCs) (Riley et al., 2019; Leko and Rosenberg, 2020). Common immunotherapies such as adoptive cellular immune therapy (ACT), blockade of immune checkpoints (ICB), and therapeutic antibodies aim to reinvigorate exhausted effector T cells and sustain immune response in solid tumors (Broz et al., 2014; Singh and McGuirk, 2020). However, the assessment of predictable benefits from immunotherapy remains a challenge. Recently, the identification of abnormally expressed genes through genetic diagnosis technology has received increased attention, in which the cytosolic DNA-sensing machinery in the tumor microenvironment (TME) emerges as a promising target to control malignant transformation and tumor progression (Vanpouille-Box et al., 2018; Kwon and Bakhoum, 2020). The cGAS–STING pathway has been discovered as an important DNA-sensing machinery in innate and adaptive antitumor immunity (Jiang et al., 2020a; Kwon and Bakhoum, 2020; Lv et al., 2020).

cGAS is one of the main DNA-sensing enzymes that can be activated by endogenous DNA and catalyze the conversion of GTP and ATP into synthesize 2-3 cyclic GMP-AMP (cGAMP) (Ablasser et al., 2013). cGAMP or cyclic dinucleotides (CDNs) binds to the downstream adaptor protein STING (stimulator of interferon gene) on the endoplasmic reticulum (ER) to activate STING (Li et al., 2013). Subsequently, activated STING migrates to the Golgi apparatus from ER to recruit and activate TBK1 kinase, follows by the downstream IRF3 or NF-kB signal (Sun et al., 2013). The cGAS-STING pathway is a vital regulator of the innate immune response during viral infections, inflammation, and anti-tumor immunity (Dhanwani et al., 2018; Lv et al., 2020; Yang et al., 2022). The tumor immunotherapy relied on the activation of the cGAS-STING pathway as tumor-derived DNA activates the tumor-infiltrating DCs (Woo et al., 2014), or tumor-derived cGAMP directly activates the STING pathway (Marcus et al., 2018), inducing the secretion of type I interferons and promoting tumor-specific antigen presentation and CTL activation (Chen et al., 2016; Du and Chen, 2018; Guan et al., 2021; Zhou et al., 2021). The activation of cGAS triggers the senescence associated secretory phenotype (SASP) which are component of proinflammatory cytokines, growth factors and chemokines (Gluck et al., 2017). Recently, cGAS–STING signal has been hypothesized as a plausible cancer suppressor during the tumor progression by causing genomic instability (Kwon and Bakhoum, 2020). Further evidence found that the cGAS-STING pathway induces inflammatory response through IL-6, IL-6R and STAT3 (Hong et al., 2022), and induces CD8^+^T cell infiltration to enhance anti-tumor immune responses in breast cancer (Pantelidou et al., 2022). The activation of STING-regulated IL-6/STAT3 increases the expression of PD-1 ligand in breast cancer (Vasiyani et al., 2022). STING agonists can synergize with anti-PD-L1 therapy to enhance the levels of IFN-β, IFN-γ, TNF-α and IL-10 as well as CD8 cytotoxic function in the breast cancer mouse model (Yin et al., 2022). With the crucial role of the cGAS-STING pathway for the crosstalk within tumor cells and immune cells close by, it is worth exploring whether cGAS-STING-related genes may provide a potential immunoregulatory mechanism in the breast tumor microenvironment (Supplementary Figure S1).

Recently, bioinformatics techniques based on high-throughput sequencing data have been widely used in the field of cancer to identify new biomarkers and construct prognostic models. The models conducted by the approaches had effective predictive power for different types of cancer, including head and neck squamous-cell carcinoma (Chi et al., 2022a), low-grade gliomas (Zhao et al., 2022) and pancreatic cancer (Chi et al., 2022b). Also, it has been widely used and valued in medicine to establish prediction model through the mechanical learning to the database (Deo, 2015; Handelman et al., 2018).

In this study, we screened 11 immune-related prognostic genes from CSRGs by integrating the RNA-seq data and clinical information from TCGA databases, and further developed a risk assessment tool based on machine learning to predict the prognostic value and evaluate the immune response in BRCA. This prognosis tool has the potential effect for informing and improving immunotherapeutic efficacy and clinical management of BRCA.

## Material and methods

### Data collection and analysis

Comprehensive information on BRCA patients was downloaded from the Genomic Data Commons Data Portal of TCGA database (https://portal.gdc.cancer.gov/), these included the RNA-seq data and clinical parameters (follow-up data, age, tumor recurrence, T and N status, pathological type and clinic stage, etc.). A total of 1087 samples were obtained from the TCGA database after eliminating the 116 samples without survival data. The inclusion criteria: (I) breast cancer patients, (II) overall survival data, (III) survival outcome data. The RNAseq information of 179 normal breast tissue samples was obtained from the GTEX database (https://gtexportal.org/). The immunohistochemistry (IHC) data were acquired from Human Protein Atlas (HPA) databases in normal breast tissues and breast tumor tissues. The RNA sequencing data and survival information on breast cancer patients of GSE20685, GSE1456, GSE3496 and GSE7390 were derived from GEO database (https://www.ncbi.nlm.nih.gov/geo/). The two published breast cancer patient cohorts GSE194040 (Wolf et al., 2022) and GSE173839 (Pusztai et al., 2021) that received anti-PD-L1 therapy were obtained from the GEO database, including gene expression data, the status of pathological complete response (pCR) and the status of MammaPrint (MP). The quantification of all RNA-seq data from the TCGA and GEO databases used a normalization method that converted the gene expression counts into transcripts per million in this study.

To establish the CSRGs risk score model, the 1080 breast cancer samples with complete clinical information were subjected to subsequent analyses, 7 samples without the complete set of all clinical information were excluded. The inclusion criteria: (I) T/N stage available, (II) clinical stage I/II/III/IV available, (III) molecular subtypes available. The exclusion criteria: (I) no T/N stage available, (II) clinical stage I/II/III/IV available, (III) no molecular subtypes available.

To verify the prognostic value for the risk score model, 15 samples without survival data from GSE3494 were also excluded. The all excluded patient samples did not be included in further analyses.

A total number of 3210 immune system pathway genes were provided from the Immunology Database and Analysis Portal (https://immport.niaid.nih.gov) and the PathCards (https://pathcards.genecards.org/) (Belinky et al., 2015). In addition, the 145 cGAS-STING-related pathway genes were obtained from the PathCards for subsequent analysis.

### Differentially expressed gene (DEG) analysis

The DEGs were identified by comparing the gene expression difference between the breast tumor tissues from the TCGA database and normal tissues from the GTEX database. The analysis was performed on the DESeq2 package using a negative binomial distribution model (Love et al., 2014), the criteria for DEG identifying were FDR < 0.05 and [log2 (fold change)] > 1. The results were displayed as volcano plots.

### Gene ontology (GO), kyoto encyclopedia of genes and genomes (KEGG) enrichment analysis and consensus clustering analysis

Functional enrichment analyses DEGs were utilized to determine the major biological characteristics. GO (http://geneontology.org/) and KEGG (https://www.kegg.jp/) analyses were based on hypergeometric distribution patterns to test the significance of functional classes in a group of differentially expressed genes. The data for the enrichment analysis were calculated using the clusterProfiler package on the R software (Yu et al., 2012). Consensus clustering analysis was performed by ConsensusClusterPlus package on R software (Wu et al., 2022).

### Construction of risk assessment and prognostic model

Based on the 35 immune-related DEGs from GSRGs, univariate Cox regression analysis was performed to identify the prognostic-related genes combined with parameters including patients’ OS, survival outcome and normalized gene expression from TCGA database, and then 11 prognostic-associated genes were selected. The significant thresholds were hazard ratio’s 95% confidence interval excluded 1 and *p* < 0.05. The Survival packages on R software were utilized in the procedure (Therneau and Grambsch, 2000).

With the application of machine learning algorithm, the filtering effect of feature variables has acquired enormous improvement, and the performance of prognostic model has been optimized (Chi et al., 2022a; Chi et al., 2022c; Chi et al., 2022d). In this study, five machine learning algorithms were integrated to construct eight composite models, as follows Elastic Net, Random Survival Forest, StepCox, CoxBoost, Gradient Boosting Machine (GBM), StepCox + GBM, Partial Least Squares Regression Cox and Survival Support Vector Machine. All models were validated on four validation datasets (GSE 1456, GSE20685, GSE3494, GSE7390). The Harrell’s concordance index (C-index) with the highest average value in validation datasets was considered as optimal model. The model building process was conducted on the glmnet package (Simon et al., 2011), randomForestSRC package (Jaeger et al., 2019), plsRcox package (Bastien et al., 2015), gbm package (Liu et al., 2022), CoxBoost package (Gonzalez-Angulo et al., 2013) and survivalsvm package (Van Belle et al., 2011) on R software.

According to the risk scoring formula of all BRCA patients from the TCGA, the median risk score was used as the cut-off point and the patients were divided into high-risk (*n* = 540) and low-risk (*n* = 540) score groups. Kaplan-Meier analysis showed the overall survival (OS) between the two risk groups until the last follow-up. Univariate and multivariate Cox analyses were used to comprehensively assess the association between risk score and patient clinical information on OS. The survminer package was utilized for the analysis (Wang et al., 2022).

### Establishment and validation of nomogram

Nomogram was constructed to predict the survival of breast cancer patients based on risk score and series clinical parameters including age, TNM (T stage, N stage, M stage) and clinical stage and age, which was calculated by the rms package (Liu et al., 2018). The predictive ability was evaluated by decision curve analysis (DCA), which was performed using the ggDCA package. Receiver operating characteristic curve (ROC) analyses were utilized to obtain area under the curve (AUC) values by using timeROC package (Blanche et al., 2013).

### Immune infiltration analysis and gene set enrichment analysis

CIBERSORTx is a tool for the deconvolution of expression matrices of human immune cell subtypes based on linear support vector regression (Newman et al., 2019). CIBERSORTx was used to evaluate the immune infiltration of breast cancer samples from the TCGA database. The ESTIMATE algorithm was used to calculate the immune, stromal, and ESTIMATE scores based on the expression of related biomarkers in immune and stromal cells (Yoshihara et al., 2013; Wu et al., 2021). The gene set enrichment analysis (GSEA; https://www.gsea-msigdb.org/gsea/index.jsp) was used to assess risk score-associated immune characteristics with annotations of immunoSigDB in the MsigDB database (Subramanian et al., 2005; Godec et al., 2016). In GSEA, the condition for identifying significant characteristics was normalized enrich score (NES) | > 1 or (NES) | < −1 and *p* < 0.05.

### Therapeutic sensitivity analysis in patients

The relationship between immunophenotype score (IPS), immune checkpoints (ICP) and risk score was estimated by Student’s *t*-test. The online tool of tumor immune dysfunction and exclusion (TIDE) algorithm (http://tide.dfci.harvard.edu/) was used to evaluate tumor immune escape, and the differences in the TIDE score between the two risk groups (Jiang et al., 2018). The machine learning algorithm One Class Linear Regression was employed to quantify the stemness of the tumor samples (Malta et al., 2018). The mRNAsi score reflected the gene expression characteristics of stem cells by analyzing the stem cell transcriptome data. The human stem cell data was provided by the Progenitor Cell Biology Consortium (PCBC) (https://www.synapse.org). The tidyverse package (Yeasmin et al., 2023) and the gelnet package (Yeasmin et al., 2023) were used to build this model.

### Drug susceptibility

To predict clinical chemotherapy responses, we build a Ridge regression model by large-scale gene expression and drug screening data. Drug sensitivity was calculated by the machine learning-based oncoPredict package based on the Genomics of Drug Sensitivity in Cancer database (GDSC) (https://www.cancerrxgene.org/), and finally obtained the 199 drugs data (Maeser et al., 2021).

### Statistical analysis

Statistical calculations were performed using Student’s *t*-test. Differences between more than two groups were calculated using ANOVA. Correlation analysis was calculated by Pearson Correlation Analysis. Data analysis and plotting were performed on R software version 4.2.2. Images were plotted by using ggplot2 package (Sun et al., 2023). NS denotes not statistically significant; *p < 0.05, **p < 0.01, ***p < 0.001 and ****p < 0.0001 indicated a statistically significant difference.

## Results

### Identification of immune-related DEGs and functional analysis in BRCA

**FIGURE 1:**
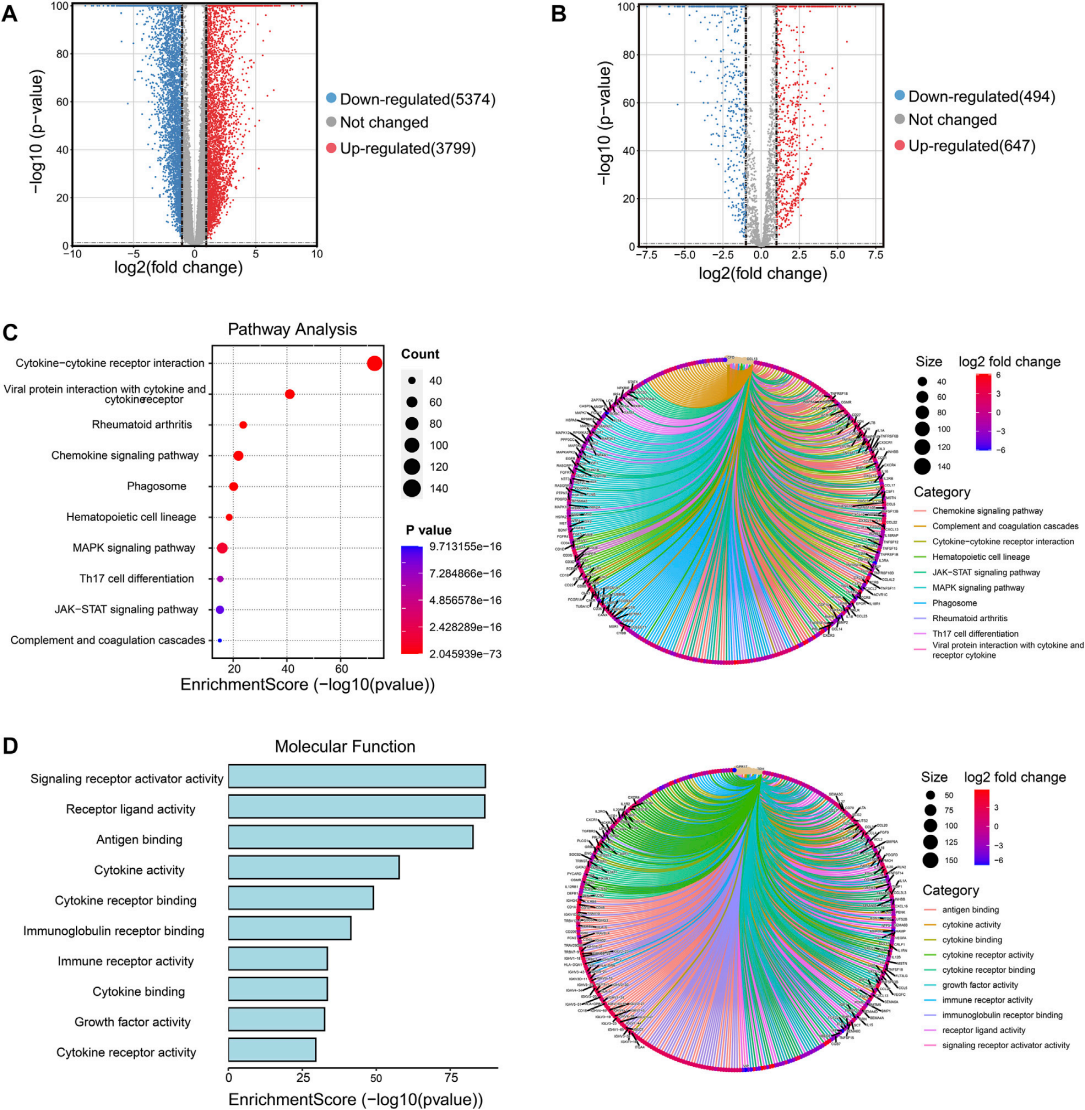
Screening the immune-related DEGs compared breast cancer tissues and normal breast tissues and identifying the functional enrichment of DEGs. **(A)** Volcano plot illustrated the 9173 DEGs between normal breast and tumor tissues, downregulated genes were 5374 and upregulated genes were 3799. Red plots represent upregulated genes and blue plots represent downregulated plots. **(B)** Volcano plot displayed the 1141 DEGs were recognized from 3210 immune-related pathway genes in normal and tumor samples, downregulated genes were 494 and upregulated genes were 647. **(C)** The top 10 pathways of 1141 DEGs were enriched and specified genes in the top 10 signal pathways were shown by a circle net diagram. **(D)** GO function analysis described the top 10 molecular functions of 1141 DEGs, and network analysis showed the representative genes and 10 biological characteristics.

Firstly, we collected the gene expression data of 179 normal breast tissue samples from the GTEx database and 1087 breast tumor samples from the TCGA database. Samples were classified into two groups according to whether they were diagnosed with breast cancer. For analyzing the DEGs in the two groups, 9173 DEGs were identified according to the pre-specified conditions of |logFC| > 1 and FDR < 0.05; the result was displayed for the volcano plot (Figure 1A). To investigate whether there were immune function-associated genes that participated in BRCA development, we next obtained 3210 genes related to the immune pathway from the ImmPort and PathCards database, the 1141 genes in them were regarded as DEGs when comparing the normal breast tissues with breast tumor tissues; the volcano plot result is charted in Figure 1B. Subsequently, the 1141 DEGs were employed to examine the functional characteristics by KEGG and GO analysis. The KEGG analysis found the enriched top 10 pathways in the 1141 DEGs included cytokine receptor, chemokines signaling, MAPK signaling, Th17 cell differentiation, and JAK/STST pathway, etc., (Figure 1C, left panel). The pathway net plot revealed the interconnections for representative genes in 10 enriched pathways (Figure 1C, right panel). The GO analysis described the significant top 10 molecular function enrichment (Figure 1D, left panel) and molecular signal characteristics (Figure 1D, right panel), these included receptor ligand activity, antigen binding, cytokine activity, etc. In summary, we identified the 1141 immune-related DEGs were crucial in breast tumor progression.

### Identification of CSRGs and functional enrichment in BRCA

**FIGURE 2:**
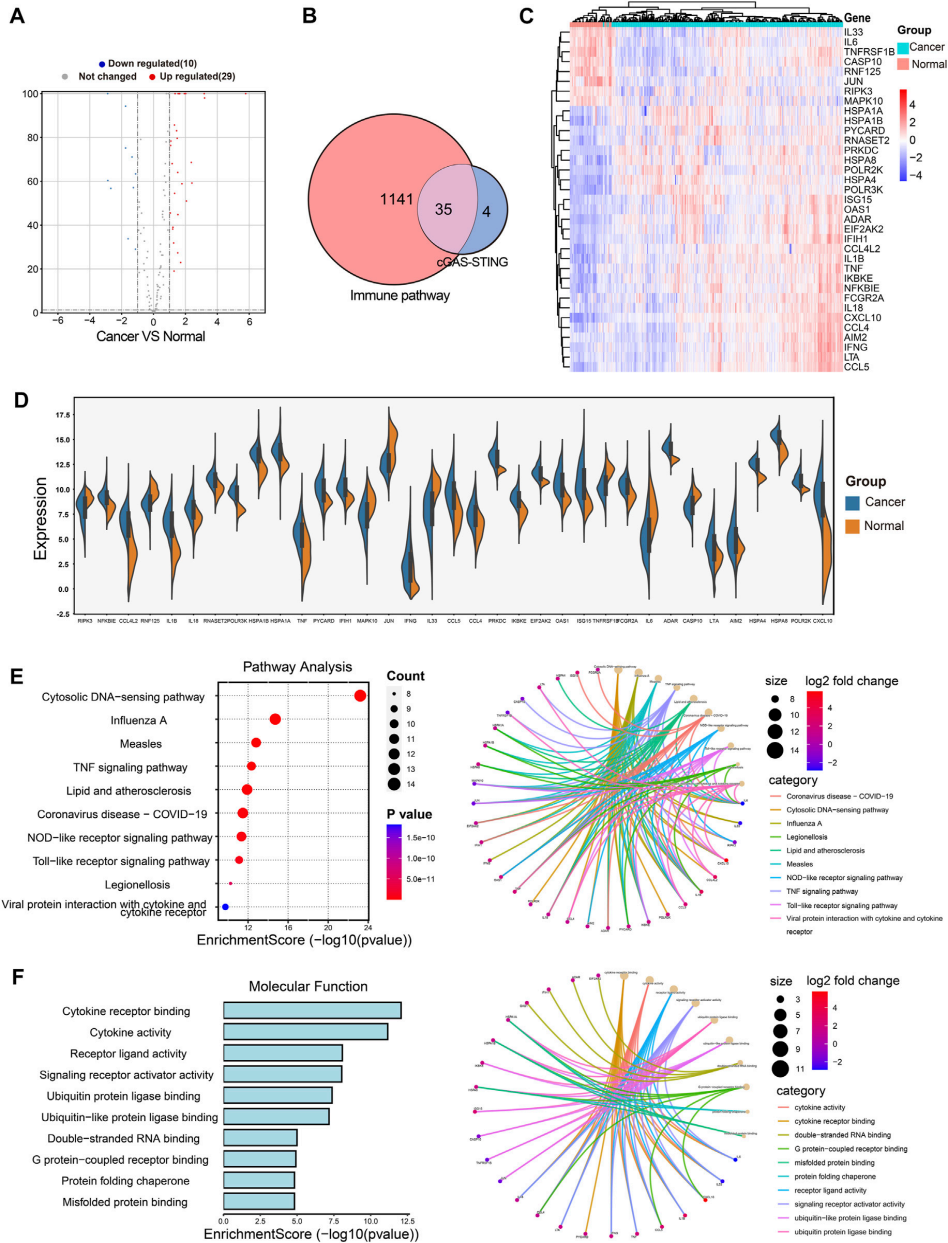
Identification of the immune-related DEGs of CSRGs in normal breast tissues and breast cancer tissues. **(A)** Volcano plot illustrated 39 DEGs of CSRGs compared normal breast tissues with breast tumor tissues. **(B)** Venn plot found the 35 DEGs of CSRGs were contained in 1141 DEGs of immune pathway-related genes. **(C)** Heat map showed the expression distribution in two groups. **(D)** The expression of 35 DEGs was displayed in a split violin diagram. **(E)** KEGG enrichment analyses showed the top 10 enriched pathway characteristics of 35 DEGs. **(F)** GO analyses described the top 10 molecular functions of 35 DEGs.

The cGAS-STING signaling is a key regulator in immune responses and plays a vital role in BRCA development and progression (Hong et al., 2022), but little is known about the role of cGAS-STING-related genes (CSRGs) in the clinical pathogenesis of breast cancer. Thus, we acquired 145 CSRGs according to the cGAS-STING gene sets from the PathCards database. We found 39 genes amongst the 145 CSRGs had significant differences in normal breast tissues and breast tumor tissues, of which 29 genes were upregulated and 10 genes were downregulated. The result was presented as a volcano plot in Figure 2A. The Venn plot in Figure 2B showed the correlation between the 39 cGAS-STING-related DEGs and the overall 1141 immune-related DEGs. Of which, 35 immune-related DEGs in CSRGs were isolated for further analysis, their expression profile in normal and tumor groups was presented as a heat map (Figure 2C), and the expression level of each DEG in the tumor and normal tissues was presented in the split violin map (Figure 2D). To understand the biological characteristics of CSRGs, functional enrichment analysis of the 35 DEGs was employed. KEGG pathway analysis showed that the top10 enriched pathway, for instance, Cytosolic DNA-sensing pathway, TNF signaling pathway, and Toll-like receptor signaling pathway, etc., these are represented on the left panel of Figure 2E. The pathway net plot demonstrated the interconnections between main pathways and their representative genes (Figure 2E, right panel). The GO analysis further revealed the top 10 molecular functions of the 35 DEGs (Figure 2F, light panel). The inter-relation between the main biological processes including ubiquitin protein ligase binding, double RNA binding, and G protein receptor combination, etc., (Figure 2F, right panel). In summary, the 35 CSRGs were identified to be associated with the BRCA immune pathway, this supported the proposition CSRGs were potential immune effectors in BRCA.

### Establishment of the risk score model based on CSRGs for predicting the prognosis of BRCA

The activation of the cGAS-STING pathway has received attention for its prognostic value in gastric cancer and hepatocellular carcinoma (Qi et al., 2020; Yang et al., 2021), it is unknown whether cGAS-STING may also be potential prognostic value in BRCA. We screened out 11 DEGs from the 35 CSRGs which were associated with the prognosis by univariate Cox regression analysis combining with patients’ OS, clinical outcome and normalized gene expression, namely, POLR2K, PYCARD, HSPA8, NFKBIE, EIF2AK2, JUN, CCL5, IL18, PRKDC, IFNG, and IL33, (Supplementary Figure S2A). The survival curves of the 11 prognostic genes in BRCA patients were evaluated by Kaplan-Meier analysis, we observed that OS was positively associated with PYCARD, NFKBIE, JUN, IL33, IL18, IFNG, and CCL5, and inversely with POLR2K, PRKDC, EIF2AK2 and HSPA8 (Supplementary Figures S3A–K). The differential expressions of the 11 prognostic genes from TCGA RNAseq data were presented in Supplementary Table S3. Then, the protein levels of the 11 genes were evaluated further using the IHC data of HPA databases in normal breast tissues and breast tumor tissues. The results showed that the expression of PYCARD, NFKBIE, PRKDC, IL18, IFNG, HSPA8, EIF2AK2, and CCL5 was increased in the human tumor tissues, IL33 and JUN performed reverse tendency, and there was no IHC data for the POLR2K (Supplementary Figures S4A–J). Next, we performed a consensus clustering analysis based on the expression level of 11 prognostic genes in 1080 patients from the TCGA database, and these patients were classified into two clusters. We found that CCL5, IFNG, IL18, IL33, JUN, NFKBIE and PYCARD had higher expression in cluster 1 (*n* = 541) compared to cluster 2 (*n* = 539), and inversely with POLR2K, PRKDC, EIF2AK2 and HSPA8 (Supplementary Figure S5A). Subsequently, 1257 DEGs were selected in two clusters for KEGG/GO analysis. We observed that the DEGs were abundant in immune-related biological characteristics and immune-related pathways (Supplementary Figures S5B, C). cGAS-STING pathway exerts important effect to enhance anti-tumor immune response and cancer biotherapy efficacy. Based on the reports in the recent decade, the molecular mechanisms of the 11 prognostic CSRGs in BRCA were displayed in Supplementary Table S1.

**FIGURE 3:**
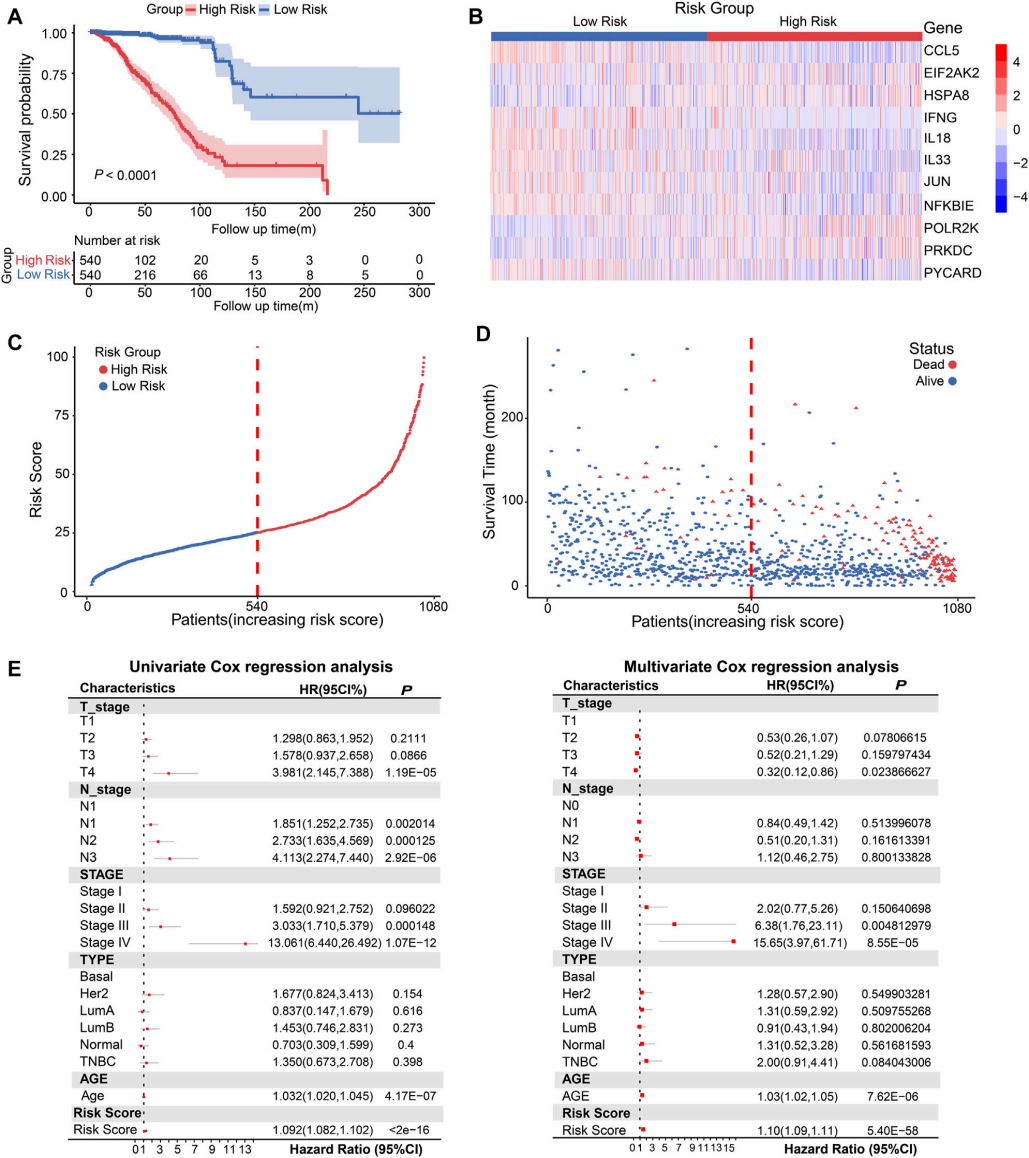
Conduction and evaluation of the CSRGs risk score model in BRCA patients. **(A)** Kaplan-Meier curve showed the OS of BRCA patients in high-risk and low-risk score groups. **(B)** Heat map presented the expression pattern of 11 prognostic-associated CSRGs in two risk score groups. **(C)** The ranked dot plot represented the risk score distribution characteristic in breast cancer patients. **(D)** Scatter plot performed survival status of patients with increasing risk score. **(E)** Forest plot of univariate and multivariate Cox regression analyses of the association between OS and clinicopathological factors (including the risk score) in BRCA patients; HR, hazard ratio; 95% CI, 95% confidence interval.

Next, we constructed eight composite models by using five machine learning algorithms in the TCGA database based on the above 11 prognosis-related genes, and further calculated the C-index of each model across training dataset and all validation datasets. The Random Survival Forest constructed by extension method of Random Forest was the optimal model in TCGA, and this model had a leading average C-index (0.70525) in all validation datasets (Supplementary Figure S6A). Then we used this model to calculate the risk score based on gene expression of 1080 breast cancer patients in TCGA database. According to the risk scores, the median risk score (25.119) was obtained, and the patients were stratified into high-risk score (*n* = 540) and low-risk score (*n* = 540) groups. The Kaplan-Meier analysis showed the patients in the low-risk group had better OS than high-risk score group (Figure 3A, *p* < 0.0001). The heatmap displayed the expression distribution for the 11 prognostic DEGs in high-risk and low-risk groups (Figure 3B), we discovered that EIF2AK2, POLR2K, PRKDC, and HSPA8 had high expressions in high-risk score, the other genes were opposite. Next, the distribution and the survival status of risk score in BRCA patients were presented in a ranked dot plot and scatter diagram, the results demonstrated a positive association between mortality and increasing risk score (Figures 3C, D).

To verify whether CSRGs risk score can act as an independent predictor of survival in BRCA, we estimated the hazard ratio (R) for OS based on risk score and a series of clinical factors including age, pathology T stage, pathology N stage, molecular subtype (Basal, Her2, luminal A, luminal B, normal and TNBC) and clinical stage (I/II/III/IV) (Supplementary Table S4). The results from univariate Cox regression analysis indicated that risk score, age, T stage, N stage, and clinical stage were significantly correlated with OS (Figure 3E, left panel). Meanwhile, when including these factors in the multivariate Cox regression analysis, the results revealed that risk score, age, clinical stage III and IV remained closely associated with the prognosis, which proved that the risk score could be used as independent factor to estimate the prognosis of BRCA patients (Figure 3E, right panel). Overall, the above evidences proved that the proposed risk model was significantly associated with survival outcomes in BRCA patients.

### Validation for the prognostic value of the CSRGs risk model

**FIGURE 4:**
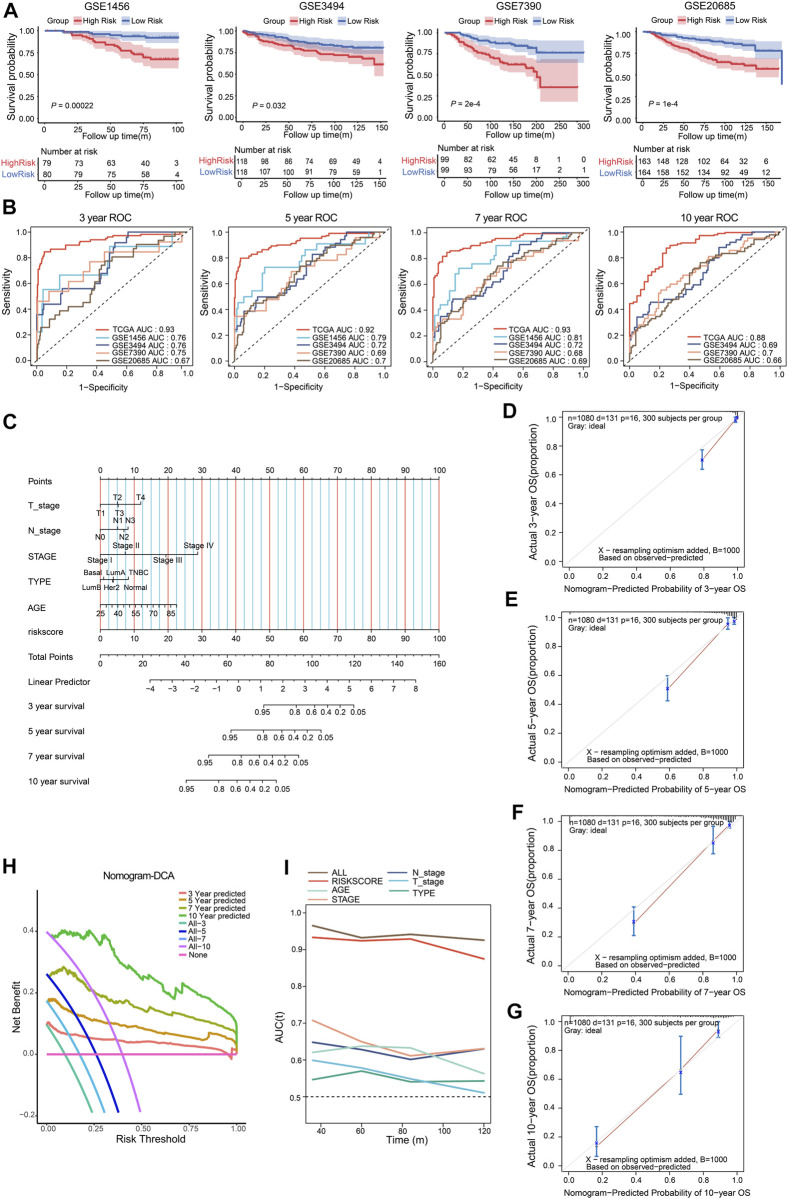
Validation of the CSRGs risk model, conduction and validation of nomogram in BRCA patients. **(A)** The Kaplan–Meier curves illustrated the survival probability between high-risk and low-risk groups in GSE1456, GSE3494, GSE7390 and GSE20685 datasets. **(B)** The ROC analysis showed the AUC for 3-, 5-, 7- and 10- year survival in the training dataset and testing datasets. **(C)** The nomogram was established by incorporating clinical parameters and the risk score to predict the probability of 3-, 5-, 7- and 10-year survival for breast cancer patients. **(D–G)** Calibration plot of the nomogram displayed the actual and predicted survival outcome for 3- **(D)**, 5- **(E)**, 7- **(F)** and 10-year **(G)**. **(H)** DCA of the nomogram for 3-, 5-, 7- and 10- year survival benefit; the straight line represents the assumption that no patients died; the wavy lines represent predictive survival; the curve lines represent actual survival. **(I)** The time-dependent ROC analyzed AUC for the predictive value compared nomogram with other parameters in BRCA patients.

To verify the prognostic value of the CSRGs risk model, the GSE1456, GSE20685, GSE3494, and GSE7390 datasets of GEO database were included in the testing cohorts. The Kaplan–Meier curve analysis showed the low-risk group performed better survival probability in GSE1456 (*p* = 0.00022), GSE3494 (*p* = 0.032), GSE7390 (*P* = 2e-4) and GSE20685 (*P* = 1e-4), indicating the survival predictive ability of the model was valid in testing datasets (Figure 4A). The ROC curves were used to further validate the prognostic accuracy of this model. We observed that the area under curve (AUC) values from the training dataset, GSE1456, GSE3494, GSE7390 and GSE20685 datasets, were 0.93, 0.76, 0.76. 0.75, and 0.67 in 3-year ROC, respectively; 0.92, 0.79, 0.72. 0.69, and 0.7 in 5-year ROC; 0.93, 0.81, 0.72, 0.68, and 0.69 in 7-year ROC; 0.88, 0.69, 0.7, and 0.66 in 10-year ROC (GSE1456 lacked 10-year survival data; Figure 4B). These data indicated that the predictive ability for risk score model was effective and reliable in BRCA patients.

To provide a quantitative method for predicting the individualized clinical prognosis on breast cancer patients, we next established a nomogram model that integrated the risk score and a series of clinical information including age, T/N stage, stage I/II/III/IV and molecular type (Basal, Normal, Her2, TNBC, Luminal A, Luminal B) in training cohort patients. The total point can be calculated in each breast cancer patient for predicting the 3-, 5-, 7- and 10-year overall survival (Figure 4C). The calibration curves for nomogram were applied to indicate the consistency, the result revealed good predictive accuracy between the practical and predicted OS for BRCA patients in 3-, 5-, 7- and 10-year (Figures 4D–G). DCA was evaluated the precision for nomogram, the results indicated that nomogram had preeminent clinical validity in predicting OS of BRCA patients (Figure 4H). Furthermore, time-dependent ROC analysis showed that the nomogram was the most accurate and powerful predictor for OS compared with other clinical features (Figure 4I), suggesting this nomogram had a good predictive power. Together, these findings validated risk score model can serve as an effective tool in predicting the prognosis of BRCA patients.

### Association of CSRGs risk score with immune score and immune cell infiltration in BRCA patients

**FIGURE 5:**
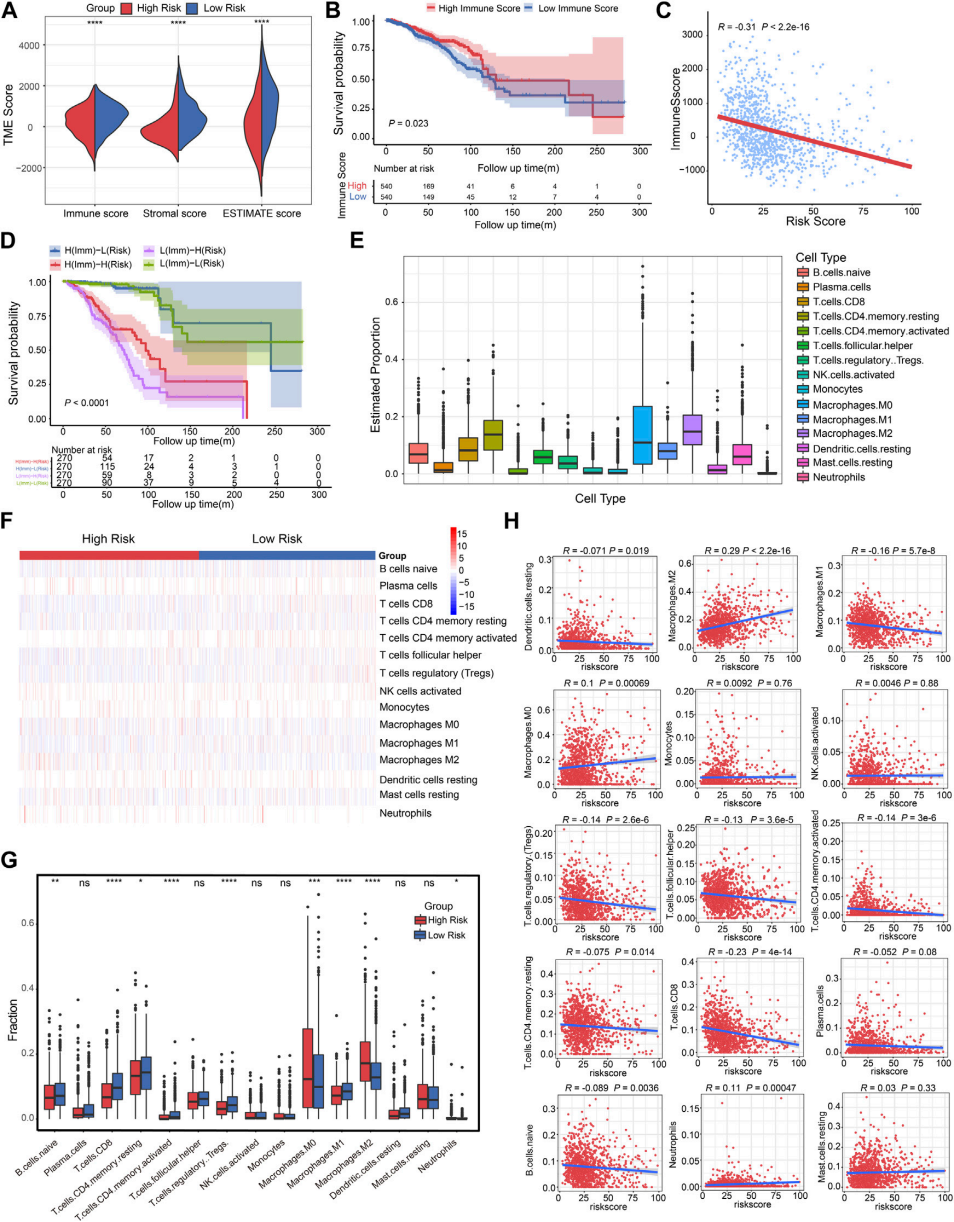
Correlation analysis of CSRGs risk score, immune score and immune cell infiltration in BRCA. **(A)** Violin plot illustrated the association between risk score and both immune and stromal scores in TME. **(B)** The Kaplan-Meier analysis was utilized to evaluate the OS between the low-immune and high-immune groups in BRCA patients. **(C)** The relationship between the immune score and risk score was displayed by box plot. **(D)** Kaplan-Meier survival analysis showed the OS among four patient groups stratified by both immune score and CSRGs risk score. **(E)** Box plots described the estimated proportion of 15 infiltrative immune cells in TME. **(F)** Heat map of the 15 infiltrative immune cells for patients in high-risk and low-risk score groups. **(G)** Box plots illustrated the different immune cell proportions in two risk score groups. **(H)** Scatter diagram showed the linear relationship between risk score and infiltrative immune cell types. *p < 0.05; **p < 0.01; ***p < 0.001; ****p < 0.0001; NS, not significant.

The complexity and diversity of the TME have an impact on immunotherapy response (Binnewies et al., 2018). Numerous studies revealed that TME status were valuable predictive indicators for tumor immune response (Zeng et al., 2019; Chen et al., 2021; Wu et al., 2021). We then used the ESTIMATE algorithm based on immune and stromal cells to calculate the TME score and further evaluate the relationship with CSRGs risk score. The split violin map demonstrated that the CSRGs risk score was strongly associated with immune score and stromal score in the TME (Figure 5A, *p* < 0.0001). To explore the impact of immune score on prognosis in BRCA patients, we also used the median value as a cut-off point to classify immune score as either high score group (*n* = 540) or low score group (*n* = 540). The Kaplan-Meier curve analysis showed the survival probability of a high-immune score was inferior to a low-immune score (Figure 5B, *p* = 0.023). Correlation analysis revealed the CSRGs risk score exhibited a negative connection with the immune score (Figure 5C, *R* = −0.31, *p* < 2.2e-16). Next, we combined the immune score and risk score to undertook further survival analysis of BRCA patients. The Kaplan-Meier analysis showed the high-immune + low-risk group had the best survival benefit, the worst survival was noted in the low-immune + high-risk group, indicating that a high-immune score and low-risk score are significant favorable prognostic indicators in BRCA (Figure 5D, *p* < 0.0001). Taken together, these analyses suggest that the CSRGs risk score and its integration with the immune score could provide a prognostic risk stratification.

Low-level infiltration of cytotoxicity immune cells can heighten tumor immune escape and impede clinical outcomes (Stanton and Disis, 2016). We then investigated the 22 human immune cell proportions in BRCA patients using the CIBERSORTx algorithm (Supplementary Figure S7A), the proportions of 15 infiltrated immune cells in TME was estimated after eliminating unobservable infiltrated immune cells (Figure 5E; Supplementary Table S5). Next, the infiltrative levels of 15 immune cells in high-risk score and low-risk score groups were displayed in a heat map, we observed that the majority of immune cells were closely related to the risk score (Figure 5F). The different proportion of these immune cells in two risk score groups was estimated, it was observed that B cells naïve, T cells CD8, T cells CD4 memory resting, T cells CD4 memory activated, T cells regulatory Tregs, Macrophages M0/M1/M2 and Neutrophils has significant difference (Figure 5G, *p < 0.05, **p < 0.01, ***p < 0.001, ****p < 0.0001). As depicted in the scatter plot (Figure 5H), the high-risk score group was positively associated with Macrophages M2, Macrophages M0 and Neutrophils; the low-risk score was correlated with elevated DC resting, Macrophages M1, T cells regulatory Tregs, T cells follicular helper, T cells CD4 memory activated, T cells CD4 memory resting, T cells CD8, and B cells naïve. Hence, we concluded that the risk score model can estimate the level of majority infiltrated immune cells for breast cancer patients.

### Immune signature analyses of GSRGs risk score in BRCA

**FIGURE 6:**
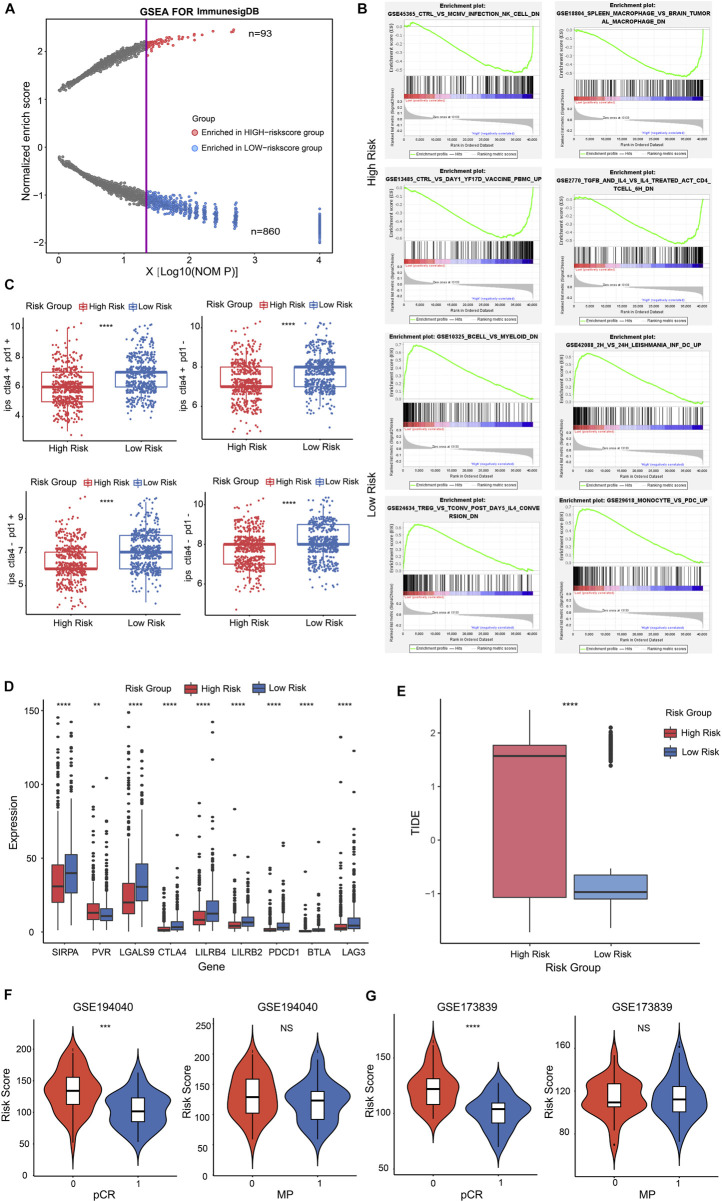
The immune signature and immunotherapy benefit of the risk score in BRCA patients. **(A)** Dot plot described the significantly enriched immune features in high-risk and low-risk groups. The red dots represented the immune features enriched in the high-risk group; blue dots represented in low-risk group. **(B)** Representatively immune characteristics in GSEA were shown in a high-risk group and a low-risk group. **(C)** The relationship was estimated between risk score and IPS score. **(D)** Box plot performed the different expression of representative ICPs in the high and low-risk groups. **(E)** Box plot showed the relationship between TIDE score and two risk score groups. **(F,G)** Violin plot illustrated the interrelation between risk score and patients’ distribution from the two pCR groups in GSE194040 cohort (F, left panel) and GSE173839 cohort [**(G)**, left panel]; pCR represented the status of a pathological complete response; 1 = complete response; 0 = failed complete response. Violin plot descripted the correlation between risk score and patients’ distribution from the two MP groups in GSE194040 cohort [**(F)**, right panel] and GSE173839 cohort (G, right panel); MP represented the MammaPrint status; 0 = MammaPrint high risk 1 (MP1); 1 = MammaPrint (ultra) high risk 2 (MP2). **p < 0.01; ***p < 0.001; ****p < 0.0001; NS, not significant.

To investigate the immune characteristics of the CSRGs risk score in BRCA patients, we performed gene set enrichment analysis (GSEA) with annotations of immunoSigDB. Significantly enriched immune features in high-risk and low-risk groups were descripted in dot plot, the results are represented in the volcano plot where the red dots represented the immune features enriched in the high-risk group (*n* = 93) while blue dots represented the low-risk group (*n* = 860) (Figure 6A; Supplementary Table S6). The significantly enriched immune features in the high-risk group such as: NK cells decreased, macrophages decreased, unstimulated peripheral blood mononuclear cells (PBMC) increased, CD3/CD28 activated CD4^+^T cells decreased (Figure 6B, upper panel); the significantly enriched immune features in the low-risk group such as: B cells decreased, dendritic cells increased, CD25^+^CD4^+^T cells decreased, monocytes increased (Figure 6B, lower panel). These findings demonstrated that risk score was associated with tumor immune response.

To investigate whether the risk score had potential effect for therapy reactiveness in BRCA patients, we next integrated the immunophenotype score (IPS) into the proposed model, the results revealed that low-risk score was positive correlation with a higher IPS score (Figure 6C, *p* < 0.0001). Next, we detected the expression of 9 representative immune checkpoints (ICPs) in BRCA patients and discovered that all ICPs were strongly associated with risk score. The result showed that the expression of PVR was positive correlation with high-risk score group, and other ICPs were positive to low-risk score group (Figure 6D, *p* < 0.01, *p* < 0.0001). By estimating the tumor immune dysfunction and exclusion (TIDE), we observed higher risk score performed a higher TIDE score (Figure 6E; Supplementary Figure S8A, *p* < 0.0001). These results indicated that low-risk score patients might be benefit from ICP inhibitors in BRCA.

Nowadays, the identification of predictive indicators is crucial for immunotherapy strategies. To investigate whether the risk score model could predict immunotherapeutic benefit for breast cancer patients, we acquired two breast cancer patient cohorts that received anti-PD-L1 therapy in GEO database. The 69 patients in the GSE194040 dataset that received Pembrolizumab (anti-PD-L1) with Paclitaxel therapy and 71 patients from the GSE173839 dataset that received Durvalumab (anti-PD-L1) with Olaparib therapy were analyzed. We next assessed the relationship between risk score and the status of pCR in patients, the results showed that patients from the complete response group (pCR = 1) had a lower risk score than failed complete response group (pCR = 0) in GSE194040 cohort (Figure 6F, *p* < 0.001) and GSE173839 cohort (Figure 6G, *p* < 0.0001). However, the risk score distribution in MP high group (MP = 0) and MP ultra-high group (MP = 1) performed not significant difference both in GSE194040 and GSE173839 (Figures 6F, G, NS). Taken together, these data implied that CSRGs risk score was related to immunotherapy responsiveness in BRCA patients, but might be unable to predict the adjunct chemotherapy decision and the risk for distant recurrence.

### Association of CSRGs risk score with subtypes, CSC score, tumor recurrence and drug analysis in BRCA patients

**FIGURE 7:**
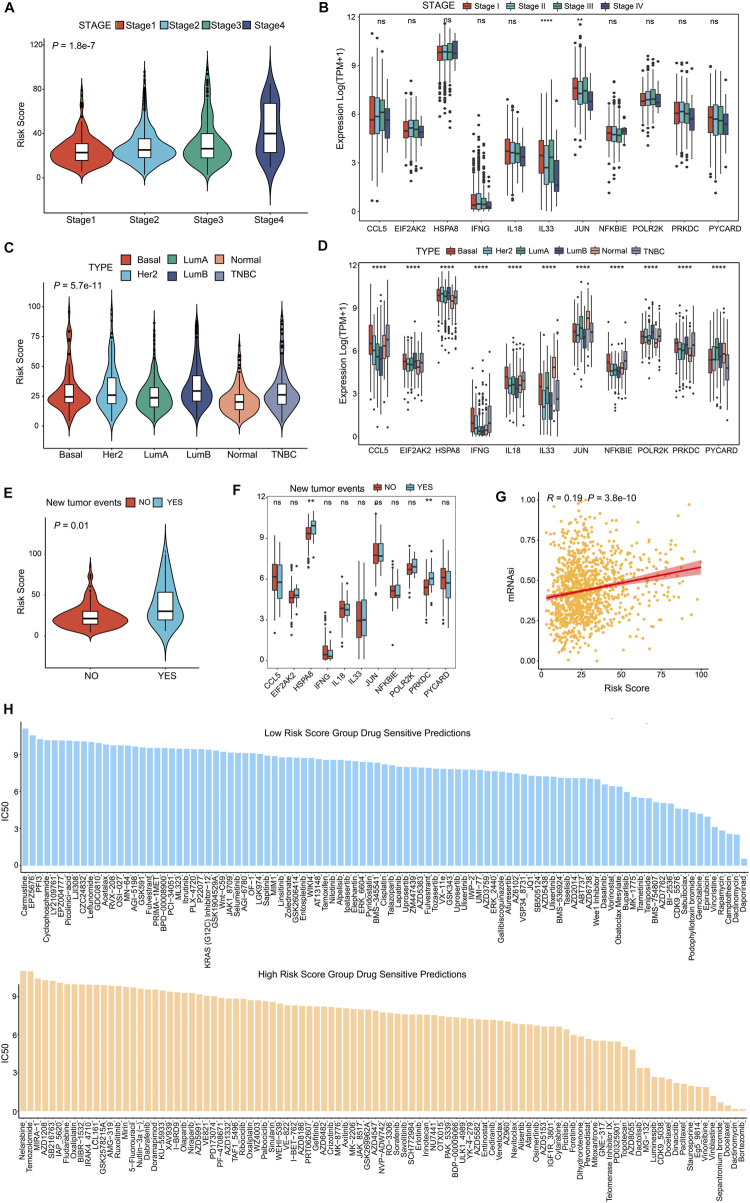
CSRGs risk score predicted clinical therapeutic benefits of BRCA patients. **(A)** Violin plot showed the association between risk score and four clinical stages (stage 1, stage 2, stage 3 and stage 4). **(B)** The different expression levels of 11 prognosis-related CSRGs in four stages was presented by a box plot. **(C)** Violin plot descripted the relationship between risk score and the molecular subtypes of Basal, Her2^+^, Luminal A, Luminal B, Normal and TNBC in BRCA patients. **(D)** The different expression levels of 11 prognosis-related CSRGs in six subtypes were described by box plot. **(E)** Violin plot displayed the correlation between the risk score and tumor recurrence in BRCA patients. **(F)** The different expressions levels of each 11 prognosis-related CSRGs in primary tumor and recurrent tumor were displayed by box plot. **(G)** CSCs detected the possibility of tumorigenesis in the low-risk score and high-score groups. **(H)** Histogram showed the drug sensibility in the low-risk score and high-score groups for BRCA patients. **p < 0.01; ****p < 0.0001; NS, not significant.

The poorer prognosis of breast cancer is affected by various factors, such as pathological grading and classification, tumor aggressiveness, therapy resistance and tumor recurrence (Phung et al., 2019; Yu et al., 2019). To identify whether the CSRGs risk score was related to clinic outcomes, we assessed the association between risk score and tumor subtype, invasive stage, tumor recurrence, cancer stem cells (CSCs) and drug sensibility. The violin plot presented in Figure 7A showed strong correlation between the four pathological stages of BRCA and the risk score. However, in the analysis of the 11 prognostic genes expression in the four BRCA stages, significant differential expression of IL33 and JUN were noted, and others had no remarkably difference (Figure 7B, NS, *p* < 0.01, *p* < 0.0001). In Figure 7C, the molecular subtype of basal, luminal A, luminal B, her2^+^, normal and triple-negative breast cancer (TNBC) were strongly associated with risk score. Next, the expression level of 11 prognostic CSRGs in the six subtypes of BRCA were analyzed, we observed that all genes performed significant difference (Figure 7D, *p* < 0.0001). Furthermore, we summarized that the risk score is strongly related to tumor recurrence in BRCA database (Figure 7E, *p* = 0.01), but only HSPA8 and PRKDC performed remarkable expression difference in the recurrent tumor and primary tumor (Figure 7F, NS, *p* < 0.01). We next utilized the cancer stem cells (CSCs) score to estimate stem cell features in BRCA so as to predict the possibility of tumorigenesis. Our results showed that the CSCs score was positively associated with increased risk score, suggesting that the BRCA cells with higher risk score had more prominent stem cell features and more likely to differentiate (Figure 7G). In addition, we investigated the potency of the risk score for forecasting the therapeutic response to chemotherapies/targeted drugs, the IC_50_ value of the 199 drugs obtained from GDSC database was estimated by the ridge regression algorithm in BRCA patients (Supplementary Table S7), the result revealed that low-risk score was positively related to Daporinad, Camptothecin, Vincristine, Epirubicin, and Trametinib, etc.; meanwhile, the high-risk score was more sensitive to Bortezomib, Dactinomycin, Staurosporine, Docetaxel and Paclitaxel (Figure 7H), these data demonstrated that risk score was associated with drug sensitivity. Taken together, we inferred that the CSRGs risk model was effective in forecasting clinic features in BRCA.

## Discussion

Over the past few decades, cGAS-STING as a center pathway of cytoplasmic DNA sensor induces a protective immune defense and provides anti-tumor immunity (Chen et al., 2016). In this research, we identified 35 differentially expressed CSRGs that were contained in immune-related DEGs of patients with BRCA. Of which, 11 CSRGs shown promise as plausible prognostic indicators in BRCA. The biological functions involved in the non-catalytic roles of cGAS include regulation of DNA repair, chronic inflammatory associated with NF-κB and MAPK pathway, cytokines signaling such as the TNF pathway, autophagy and lysosome-dependent cell death (Qi et al., 2020; Zhang et al., 2020). Consistently, our KEGG and GO analysis demonstrated that CSRGs were markedly enriched in cytosolic DNA-sensing pathway, cytokines activity, and cytokine receptor binding, etc. In our proposed risk model, the low-risk group had a lower hazard ratio and mortality than the high-risk group. The nomogram model could accurately predict the 3-, 5- and 10-year survival probability of individual BRCA patients, and the internal and external validation further affirmed the creditability. These results strongly suggested that CSRGs risk score was identified as a potential prognostic indicator and provide a promising strategy for improving clinic outcomes in BRCA patients.

Here, we reported the cGAS-STING-related genes have strong correlation with immune cells infiltration and immune checkpoints expression in BRCA. Recent evidence indicates that the various immunotherapy effect is dependent upon the activation of cGAS-STING-related genes in BRCA. Some genes involved in our risk model have been reported. IL33 facilitates immunosuppressive (Jovanovic et al., 2014), and fibroblast-derived IL33 modifies the immune microenvironment to promote breast cancer growth and metastases (Shani et al., 2020). Induction or recombinant IL-33 enhances immune checkpoint blockade treatment in breast cancer (Blomberg et al., 2023). IFN-γ-driven immunosuppressive pathway has been a target in MUC1-C alone or combination with ICPs treatment for TNBC (Yamashita et al., 2021). HSPA8 is a new biomarker related to prognosis and immune infiltration in TNBC (Ying et al., 2022). CCL5 mediates breast tumor progression and recurrence by interacting with the CCR3 axis (Yamaguchi et al., 2021), recruiting macrophages (Walens et al., 2019) and regulating the CD4^+^/CD8^+^, CCR5^+^/CD4^+^ or Treg/CCR5^+^ cell ratios (Qiu et al., 2022). IFI16-dependent STING pathway can potentiate HER2 breast cancer responses to immunotherapy (Ong et al., 2022). Considering these findings, the CSRGs will be potential useful biomarkers to provide novel therapeutic strategy for breast cancer.

CSRGs score was related to the clinicopathological features of BRCA in our study. For instance, the clinical stages and molecular subtype of BRCA had powerful correlation with risk score. The clinical stage I patients had lower risk score and stage IV performed the highest risk score. In addition, the risk of BRCA recurrences was strongly correlated with the original TNM status (Pan et al., 2017). Whilst the T cell infiltration of patients with early cancer progress may help control tumor recurrence (Mlecnik et al., 2011). Due to their self-renewal capacity and potential differentiative capacity, CSCs was associated with tumor progression, metastasis, treatment resistance, and poor prognosis (Ding et al., 2020). In our study, higher risk score was positively associated with tumor recurrence and stem cell characteristics, inferring that patients in the high-risk score may be response worse to existing therapies.

Evaluating the TME status may predict the prognosis of patients and may be used as a biomarker for immunotherapy (Wu et al., 2021). We calculated TME scores and found that the low-risk score obviously presented higher immune and stromal scores. Immune interaction is critical characteristic of tumorigenesis and prognosis, and we discovered that the immune score performed survival prediction ability and the higher-immune score had superior OS in BRCA patients. The prognostic value of immunophenotype may be more powerful than the traditional staging (Azimi et al., 2012; Gajewski et al., 2013). PD1/PD-L1 expression and immune checkpoint therapy have been regarded as important indicators for clinical guidance and represent immunotherapy responsiveness (Sharpe and Pauken, 2018; Liu et al., 2021). However, many challenges still exist of immune checkpoint therapy for cancers due to the low response rate and immune-related adverse events in some cancer patients (Darvin et al., 2018). Our results indicated that CSRGs risk score was highly correlated to immune checkpoint scores, including PD1, SIRPA, and LAG4. Moreover, risk score performed association with immunotherapy response in breast cancer immunotherapy cohort. The immunotherapy efficacy depends on the infiltration of effector CD8^+^T cells and the presence of tumor-associated macrophages in TME (DeNardo and Ruffell, 2019; Jiang et al., 2020b). But the majority of patients show unsatisfied immunotherapies due to the exhausted and dysfunctional state of immune T cells in the clinic (Jiang et al., 2020b). Our results discovered that B cells, CD8 T cells, helper T cells, resting DC cells, monocytes and M1 macrophages were positively associated with the low-risk group. Consequently, the low-risk patients may benefit more from immunotherapy, and it may greatly help to improve immunotherapy while reducing its immune-suppressive effects. Therefore, the cGAS-STING pathway may provide new approaches to enhance immunotherapy in breast cancer.

Without a doubt, there are still some limitations in this study. The prognostic model was conducted based on the publicly accessible data, and there was lacking strong support of laboratory and clinical data. So, it would be necessary to further validate the practical value of the model through animal and cell experimental studies, and even larger follow-up studies. In particular, the biological functions of the 11 genes associated with cGAS-STING pathway in breast cancer need to be further elucidated and assessed.

## Conclusion

In summary, we constructed a new risk score model using machine learning arithmetic based on 11 prognostic-related CSRGs (POLR2K, PYCARD, HSPA8, NFKBIE, EIF2AK2, JUN, CCL5, IL18, PRKDC, IFNG, IL33) to effectively predict prognosis and immunotherapy benefits in breast cancer patients. Favorable performance in validation datasets suggested its believable perspective in utilization.

## Data Availability

The datasets presented in this study can be found in online repositories. The names of the repository/repositories and accession number(s) can be found in the article/Supplementary Material.
