# Supplementary material for: Development of a risk model to predict prognosis in breast cancer based on cGAS-STING-related genes
Source: Front Genet. 2023 Mar 27;14:1121018. doi: 10.3389/fgene.2023.1121018 (PMC10083333; doi:10.3389/fgene.2023.1121018)
Supplement: Supplementary file 2 [file Table1.docx]

Supplementary Material

Development of a risk model to predict prognosis in breast cancer based on cGAS-STING-related genes

Chen Chen*, Junxiao Wang, Chao Dong, David Lim

*** Correspondence:** Zhihui Feng: fengzhihui@sdu.edu.cn

1. **Supplementary Figures**

**
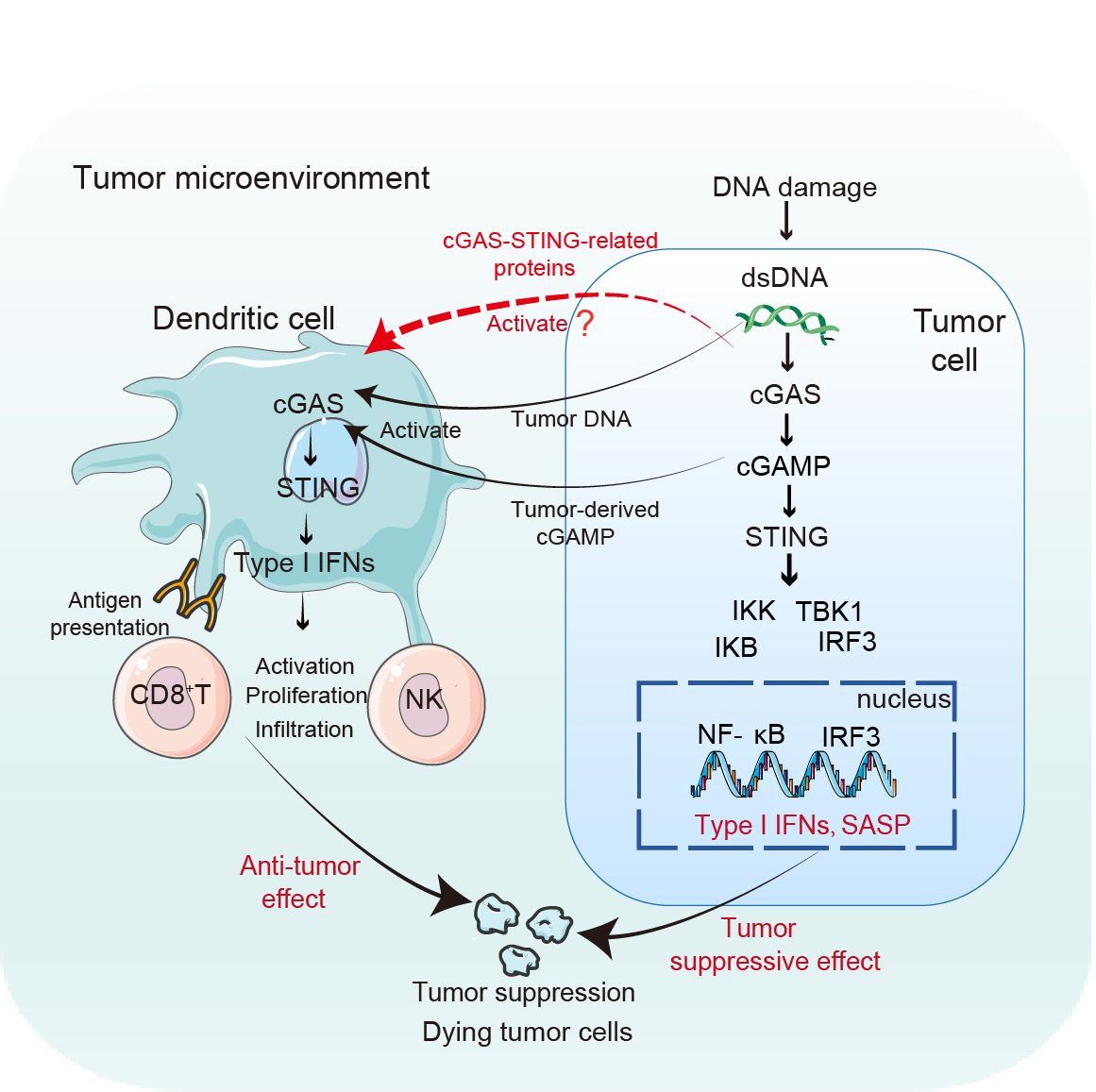
**

**Supplementary Figure 1**. A diagram illustrated the cGAS-STING pathway plays a crucial role in cancer-immunity cycle. In tumor cells, the aberrant DNA accumulation generated from different sources can stimulate cGAS-STING pathway, induced the secretion of type I IFNs and SASP to promote tumor suppression. In addition, tumor-derived cGAMP or DNA induced the activation of cGAS-STING pathway in DCs, which upregulated the secretion of type I IFNs and enhanced the activation of CD8 /NK cells to mediate anti-tumor response. Based on the crucial role of the cGAS-STING pathway for the crosstalk with tumor cells and immune cells nearby, we hypothesized that the cGAS-STING-related genes or proteins may also provide potential immunoregulatory effect in breast tumor microenvironment.

**
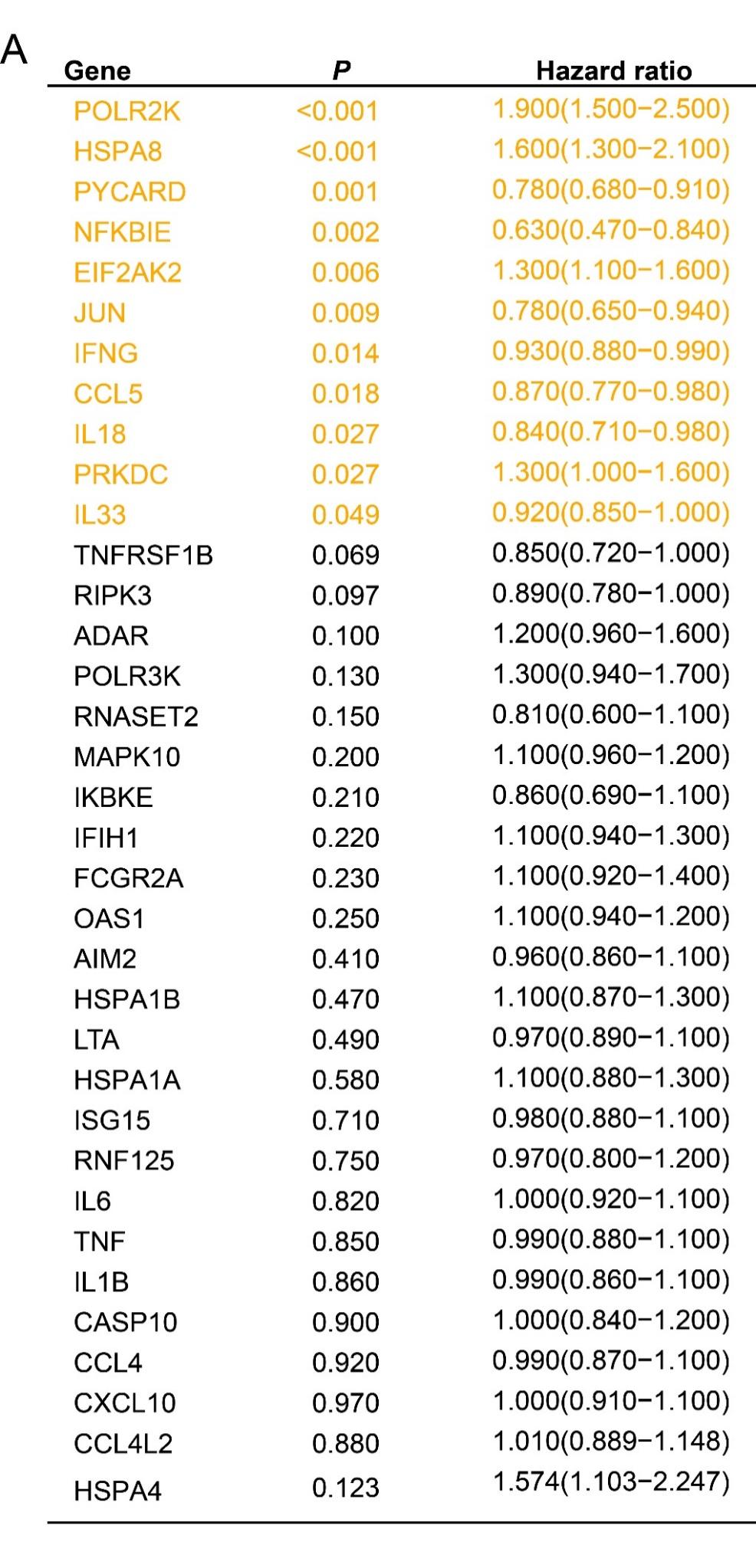
**

**Supplementary Figure 2**. Identifying the 11 prognostic-related genes in BRCA. **(A)** The univariate Cox regression analysis estimated the HR for OS based on the 35 DEGs of CSRGs. Hazard ratio’s 95% CI excluded 1 and *P* < 0.05 are regarded as conditions.

**
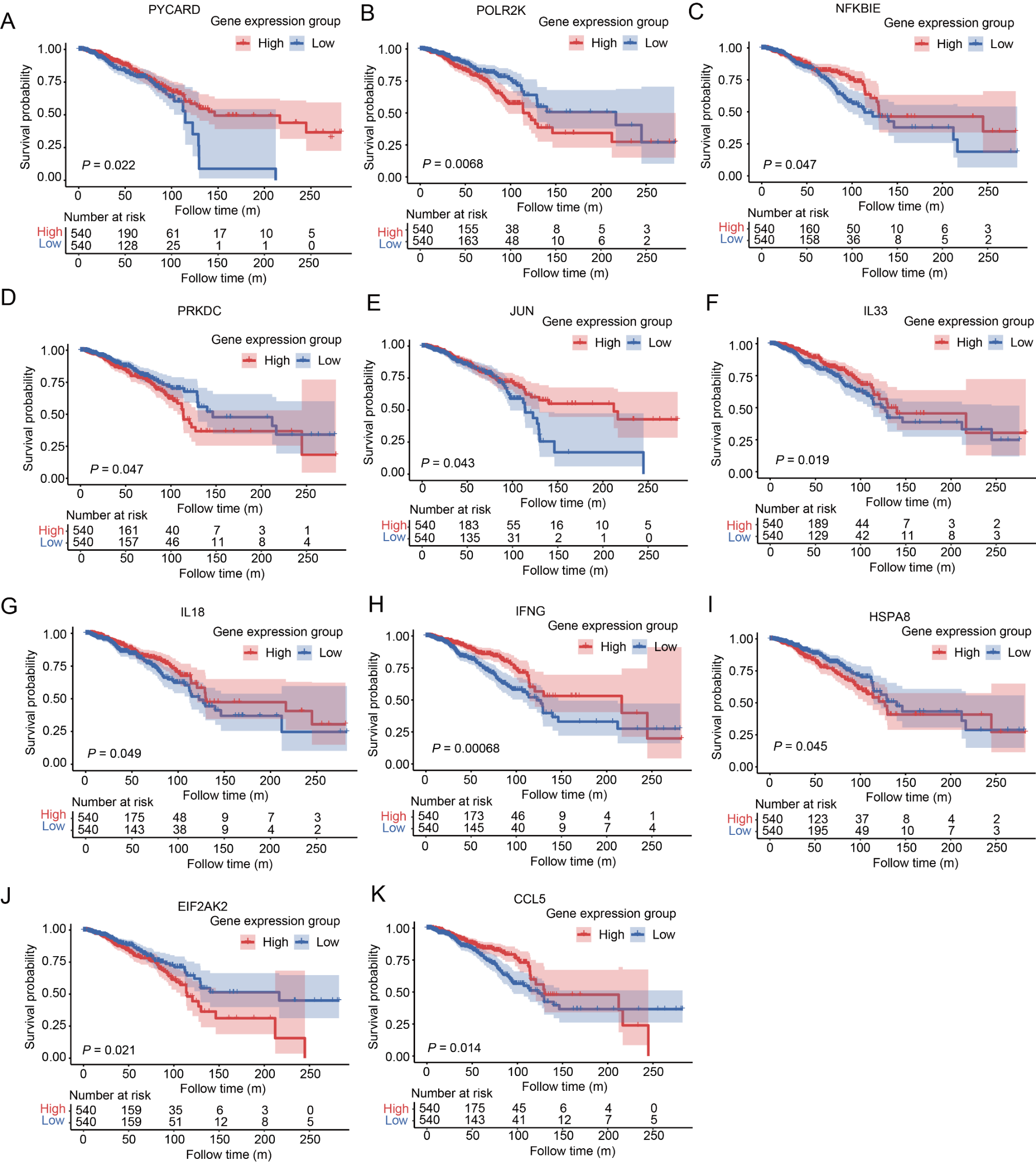
**

**Supplementary Figure 3**. The association between the 11 prognostic DEGs with OS in patients from the TCGA dataset. **(A-K)** Kaplan–Meier analysis presented the independent survival curve of PYCARD (A), POLR2K (B), NFKBIE (C), PYKDC (D), JUN (E), IL33 (F), IL18 (G), IFNG (H), HSPA8 (I), EIF2AK2 (J), CCL5 (K).

**
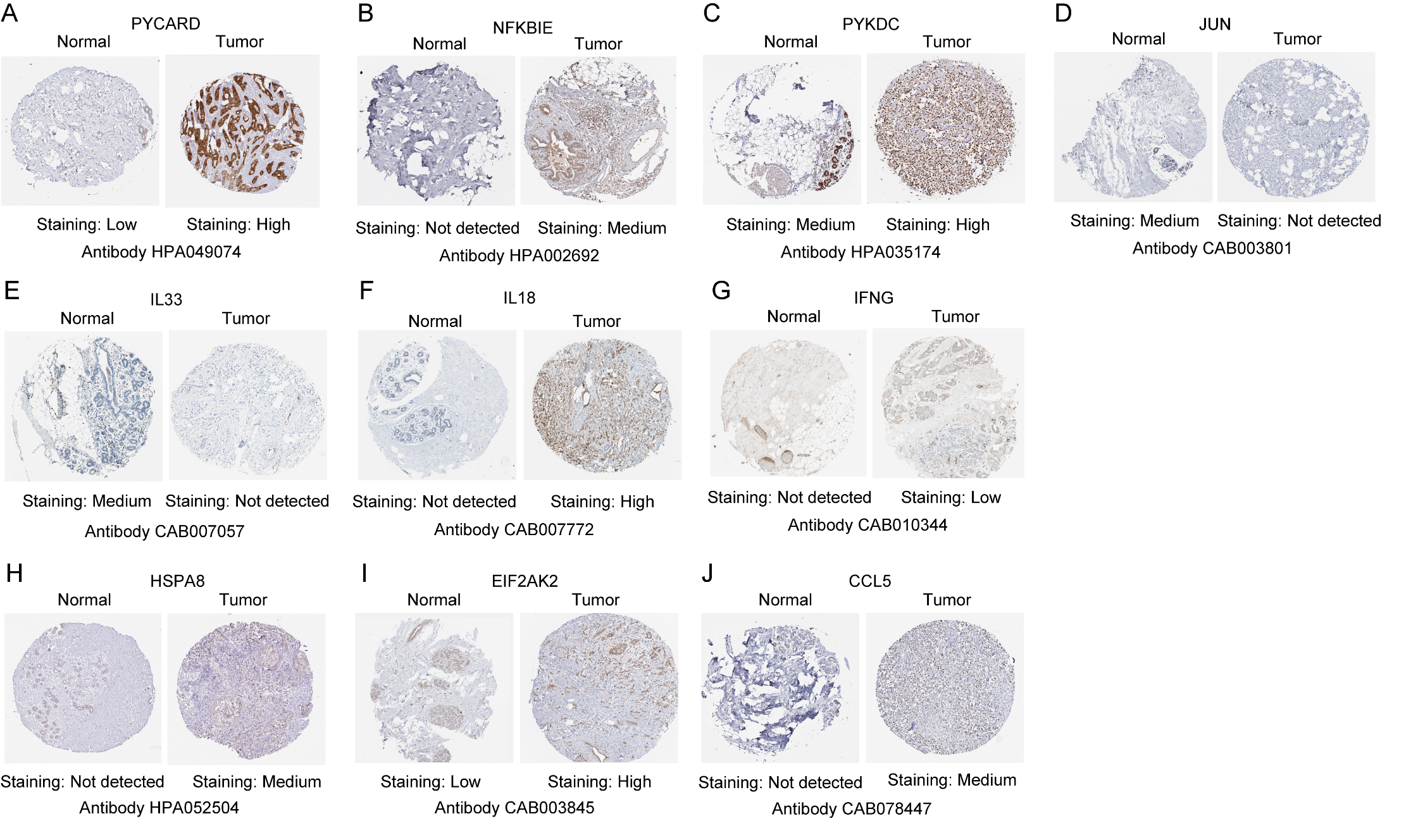
Supplementary Figure 4**. The differential expressions of the 10 prognostic DEGs besides POLR2K in breast normal and tumor tissues. **(A-J)** Representative IHC staining images from the HPA database showed the protein levels of PYCARD (A), NFKBIE (B), PYKDC (C), JUN (D), IL33 (E), IL18 (F), IFNG (G), HSPA8 (H), EIF2AK2 (I), CCL5 (J).


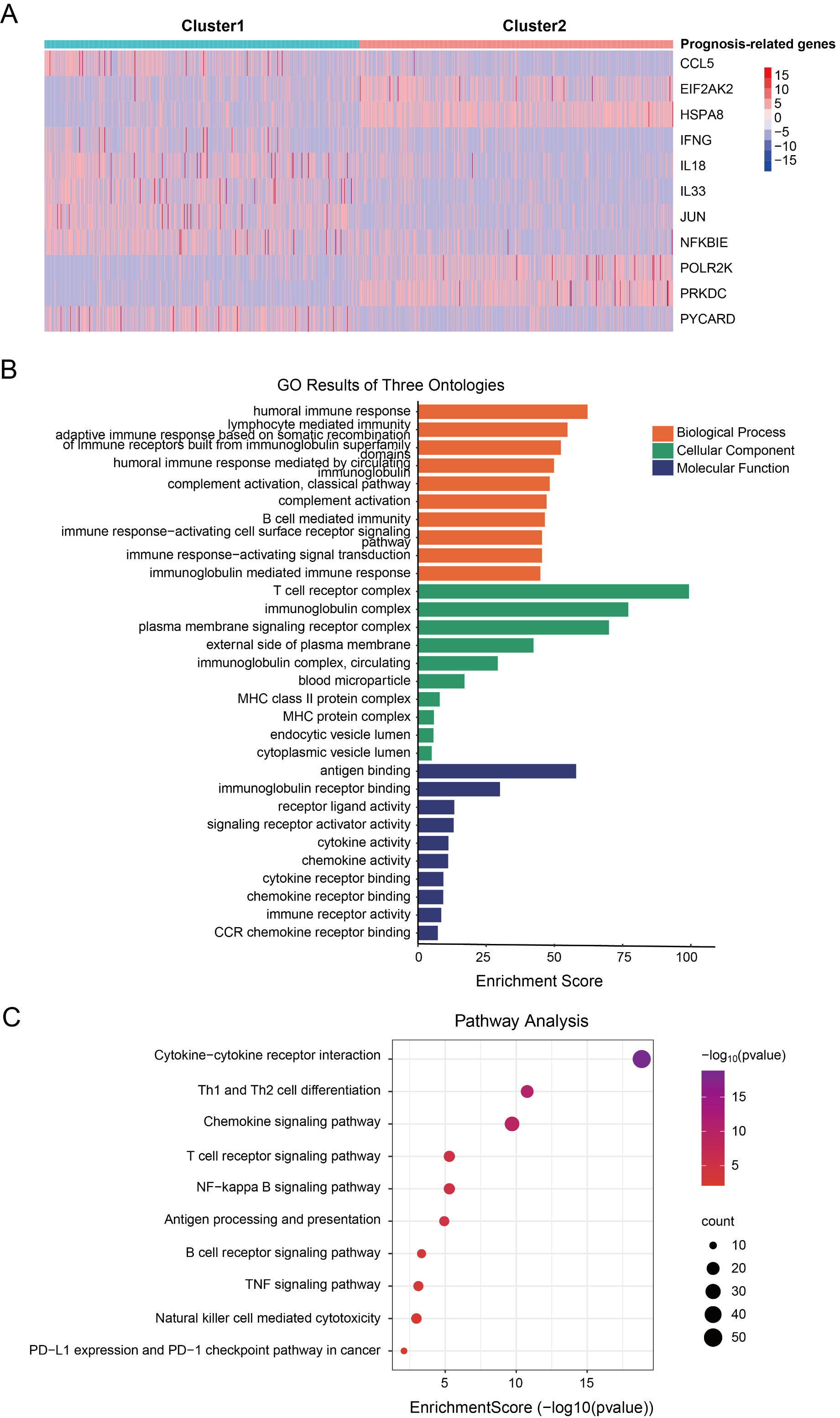


**Supplementary Figure 5.** The consensus clustering analysis based on the expression level of 11 prognostic genes in 1080 patients from TCGA database. **(A)** Heat map showed the expression level of 11 prognostic genes in the two clusters. **(B-C)** KEGG/GO analysis enriched the top 10 biological process, cellular component, molecular function and pathway of DEGs in the two clusters.


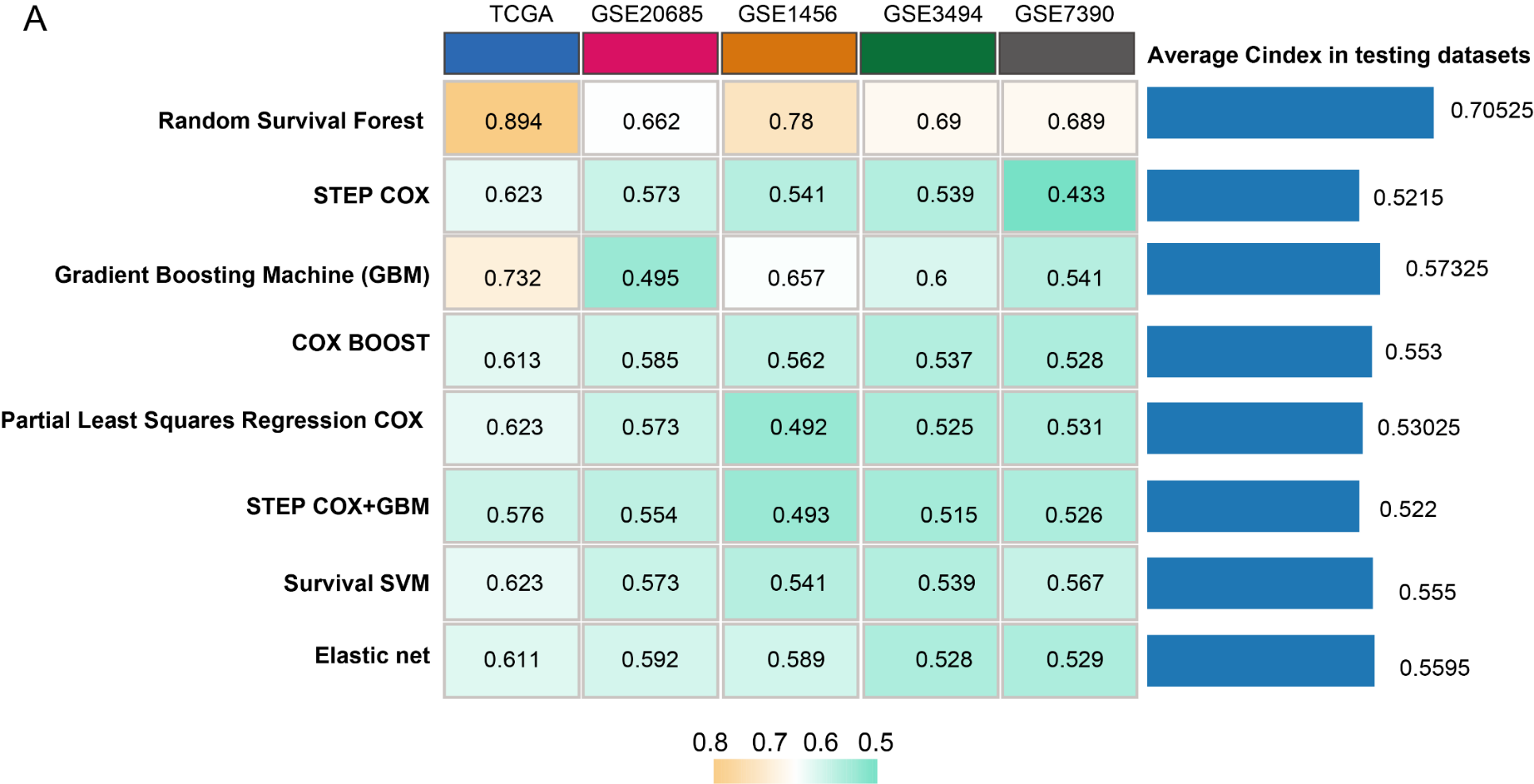


**Supplementary Figure 6.** Construction of the CSRGs prediction model via the machine learning-based integrative procedure. **(A)** The C-index of eight composite models in training dataset and testing datasets.

**
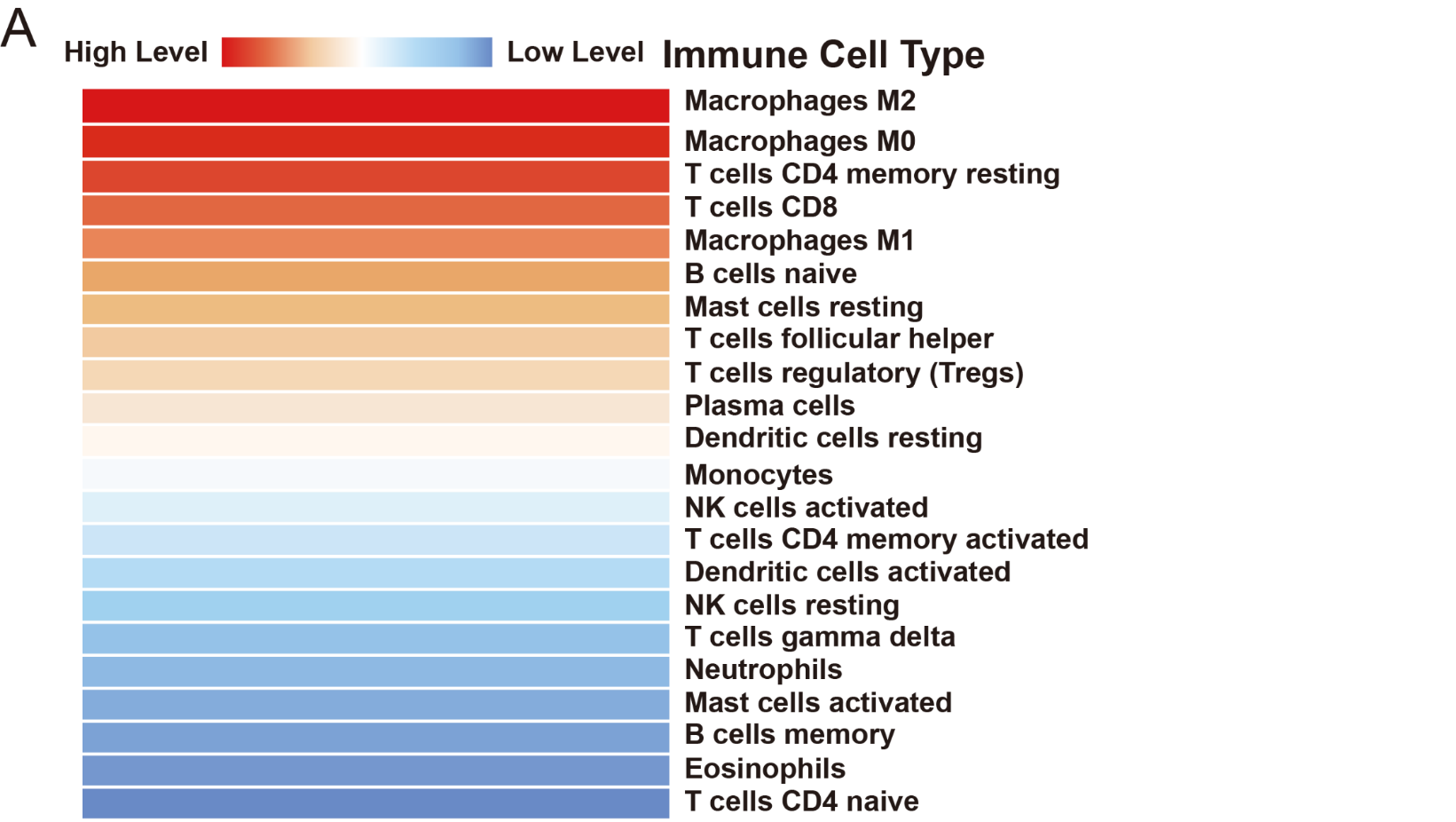
**

**Supplementary Figure 7**. The identification of infiltrating immune cells in TME of BRCA. **(A)** The infiltrated proportion of the 22 human immune cell subpopulations was assessed by using the CIBERSORTx algorithm.

**
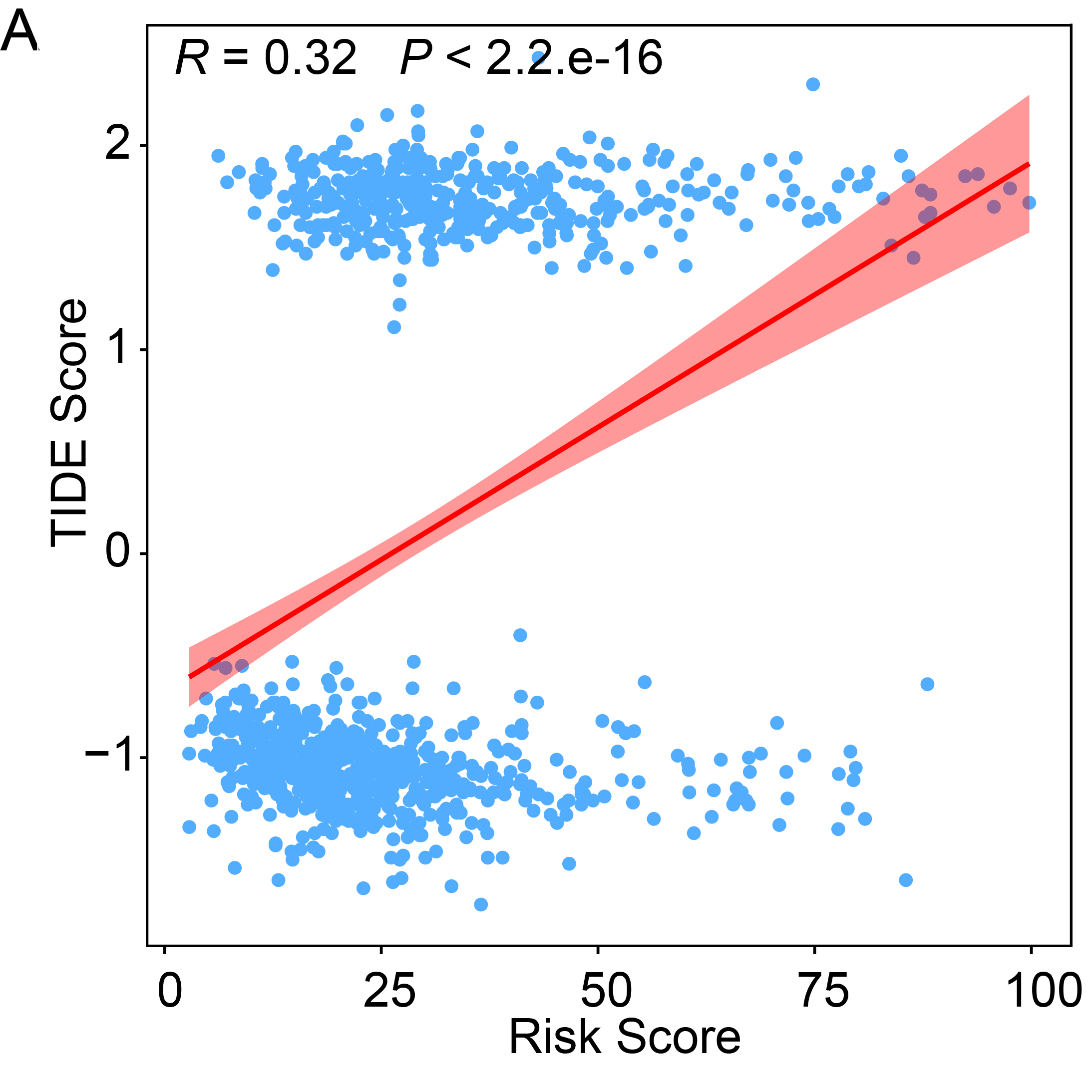
**

**Supplementary Figure 8.** Estimating the interrelation between risk score and tumor immune dysfunction and exclusion. **(A)** Scatter diagram displayed the linear relation of TIDE score and risk score in breast cancer.

1. **Supplementary Table**

Supplementary Table 1. The mechanism and function of the 11 cGAS-STING related genes

| **Gene** | **Mechanism and Function** |
| --- | --- |
| POLR2K | POLR2K is a cellular important antioxidant signal [1], associates with the transport of respiratory electrons and signaling NGF from PPI network [2]. Studies reported POLR2K is related to immunotherapy of breast cancer by using molecular descriptors and artificial neural networks [3]. |
| PYCARD | PYCARD codes for the ASC adaptor protein required for assembly of the majority of canonical inflammasomes [4], plays important role in breast cancer including immune modulation [5], DNA methylation levels [6], apoptosis regulation [7]. |
| HSPA8 | HSPA8 represents extracellular vesicles and particles (EVPs) marker [8], associates with poor metastasis-free survival [8] and early relapse [9] in breast cancer. |
| NFKBIE | NFKBIE is a negative regulator of NF-κB [10, 11]. The mutant variants of NFKBIE activate the TNFα/NF-κB pathway and contributes to a favorable anti-PD1 treatment response in mutational [10]. NFKBIE also as immune-related gene predicts breast cancer patient survival [12, 13]. |
| EIF2AK2 | Eukaryotic translation initiation factor 2α kinase 2 (EIF2AK2) is one of the 4 mammalian kinases [14] and activated by double-stranded RNA (dsRNA) during viral infections [15], which has been reported that suppresses the metastatic capabilities of several types of cancer cells, including breast cancer [16]. |
| JUN | JUN (c-Jun, Jun-B, and Jun-D) is a major component of the heterodimeric transcription factor AP-1 [17, 18]. c-Jun regulates migration and invasion [19] and relates to endocrine therapies and tumorigenesis [20] in breast cancer. |
| CCL5 | Chemokine CCL5 plays a crucial role in breast cancer tumorigenesis and progression [21], which participates in the recruitment of immune cells, induces immunosuppression and favoring tumor progression [21, 22]. |
| IL18 | IL-18 activates cytotoxic T cells (CTLs) or NK cells to produce IFN-γ and contribute to tumor immunity [23]. In breast cancer, the increase of IL18 level controls tumor progression [24], influences YAP1 expression via IFN-γ production. It also has positive association with clinical outcome [25]. |
| IL33 | IL-33 exerts pro-tumorigenesis in various cancers [26]. For instance, IL33 induces endocrine resistance of breast cancer by promoting cancer stem cell properties [27], promotes breast cancer growth and metastases by facilitating intra-tumoral accumulation of immunosuppressive and innate lymphoid cells [28]. Induction or recombinant IL-33 promotes immune checkpoint blockade (ICB) treatment in breast cancer [29]. |
| PRKDC | PRKDC has been regarded as a new biomarker and drug target for ICB immunotherapy [30]. PRKDC participates in the DNA damage response [31], regulates chemosensitivity [32] and mediated p38-MAPK signaling [33] in breast cancer, and high expression of PRKDC is associated with poor survival of breast cancer patients [33]. |
| IFNG | IFNG (IFN-γ) signaling augments immune function and antagonizes both T cell and innate immune responses in various tumor cells [34]. IFN-γ as ferroptosis-associated gene predict breast cancer prognosis [35]. High enriched CD8 cells enhance IFN-γ response and associate with better survival in TNBC [36]. |

**References**

[1] C. Pang, Y.C. Sheng, P. Jiang, H. Wei, L.L. Ji, Chlorogenic acid prevents acetaminophen-induced liver injury: the involvement of CYP450 metabolic enzymes and some antioxidant signals, J Zhejiang Univ Sci B, 16 (2015) 602-610.

[2] P. Devarbhavi, L. Telang, B. Vastrad, A. Tengli, C. Vastrad, I. Kotturshetti, Identification of key pathways and genes in polycystic ovary syndrome via integrated bioinformatics analysis and prediction of small therapeutic molecules, Reprod Biol Endocrinol, 19 (2021) 31.

[3] A. Lopez-Cortes, A. Cabrera-Andrade, J.M. Vazquez-Naya, A. Pazos, H. Gonzales-Diaz, Y.M.C. Paz, S. Guerrero, Y. Perez-Castillo, E. Tejera, C.R. Munteanu, Prediction of breast cancer proteins involved in immunotherapy, metastasis, and RNA-binding using molecular descriptors and artificial neural networks, Sci Rep, 10 (2020) 8515.

[4] B. Ritchey, Q. Hai, J. Han, J. Barnard, J.D. Smith, Genetic variant in 3' untranslated region of the mouse pycard gene regulates inflammasome activity, Elife, 10 (2021).

[5] B.Z. Fite, J. Wang, A.J. Kare, A. Ilovitsh, M. Chavez, T. Ilovitsh, N. Zhang, W. Chen, E. Robinson, H. Zhang, A. Kheirolomoom, M.T. Silvestrini, E.S. Ingham, L.M. Mahakian, S.M. Tam, R.R. Davis, C.G. Tepper, A.D. Borowsky, K.W. Ferrara, Immune modulation resulting from MR-guided high intensity focused ultrasound in a model of murine breast cancer, Sci Rep, 11 (2021) 927.

[6] L. Hamadneh, M. Al-Majawleh, Y. Jarrar, S. Shraim, M. Hasan, B. Abu-Irmaileh, Culturing conditions highly affect DNA methylation and gene expression levels in MCF7 breast cancer cell line, In Vitro Cell Dev Biol Anim, 54 (2018) 331-334.

[7] M. Sharma, Apoptosis-antagonizing transcription factor (AATF) gene silencing: role in induction of apoptosis and down-regulation of estrogen receptor in breast cancer cells, Biotechnol Lett, 35 (2013) 1561-1570.

[8] A. Hoshino, H.S. Kim, L. Bojmar, K.E. Gyan, M. Cioffi, J. Hernandez, C.P. Zambirinis, G. Rodrigues, H. Molina, S. Heissel, M.T. Mark, L. Steiner, A. Benito-Martin, S. Lucotti, A. Di Giannatale, K. Offer, M. Nakajima, C. Williams, L. Nogues, F.A. Pelissier Vatter, A. Hashimoto, A.E. Davies, D. Freitas, C.M. Kenific, Y. Ararso, W. Buehring, P. Lauritzen, Y. Ogitani, K. Sugiura, N. Takahashi, M. Aleckovic, K.A. Bailey, J.S. Jolissant, H. Wang, A. Harris, L.M. Schaeffer, G. Garcia-Santos, Z. Posner, V.P. Balachandran, Y. Khakoo, G.P. Raju, A. Scherz, I. Sagi, R. Scherz-Shouval, Y. Yarden, M. Oren, M. Malladi, M. Petriccione, K.C. De Braganca, M. Donzelli, C. Fischer, S. Vitolano, G.P. Wright, L. Ganshaw, M. Marrano, A. Ahmed, J. DeStefano, E. Danzer, M.H.A. Roehrl, N.J. Lacayo, T.C. Vincent, M.R. Weiser, M.S. Brady, P.A. Meyers, L.H. Wexler, S.R. Ambati, A.J. Chou, E.K. Slotkin, S. Modak, S.S. Roberts, E.M. Basu, D. Diolaiti, B.A. Krantz, F. Cardoso, A.L. Simpson, M. Berger, C.M. Rudin, D.M. Simeone, M. Jain, C.M. Ghajar, S.K. Batra, B.Z. Stanger, J. Bui, K.A. Brown, V.K. Rajasekhar, J.H. Healey, M. de Sousa, K. Kramer, S. Sheth, J. Baisch, V. Pascual, T.E. Heaton, M.P. La Quaglia, D.J. Pisapia, R. Schwartz, H. Zhang, Y. Liu, A. Shukla, L. Blavier, Y.A. DeClerck, M. LaBarge, M.J. Bissell, T.C. Caffrey, P.M. Grandgenett, M.A. Hollingsworth, J. Bromberg, B. Costa-Silva, H. Peinado, Y. Kang, B.A. Garcia, E.M. O'Reilly, D. Kelsen, T.M. Trippett, D.R. Jones, I.R. Matei, W.R. Jarnagin, D. Lyden, Extracellular Vesicle and Particle Biomarkers Define Multiple Human Cancers, Cell, 182 (2020) 1044-1061 e1018.

[9] Y. Min, Y. Feng, H. Luo, D. Hu, X. Wei, D. He, G. Yin, S. Fan, Identifying and Validating of an Autophagy-Related Gene Signature for the Prediction of Early Relapse in Breast Cancer, Front Endocrinol (Lausanne), 13 (2022) 824362.

[10] C.M. Amato, J.D. Hintzsche, K. Wells, A. Applegate, N.T. Gorden, V.M. Vorwald, R.P. Tobin, K. Nassar, Y.G. Shellman, J. Kim, T.M. Medina, M. Rioth, K.D. Lewis, M.D. McCarter, R. Gonzalez, A.C. Tan, W.A. Robinson, Pre-Treatment Mutational and Transcriptomic Landscape of Responding Metastatic Melanoma Patients to Anti-PD1 Immunotherapy, Cancers (Basel), 12 (2020).

[11] L. Mansouri, L.A. Sutton, V. Ljungstrom, S. Bondza, L. Arngarden, S. Bhoi, J. Larsson, D. Cortese, A. Kalushkova, K. Plevova, E. Young, R. Gunnarsson, E. Falk-Sorqvist, P. Lonn, A.F. Muggen, X.J. Yan, B. Sander, G. Enblad, K.E. Smedby, G. Juliusson, C. Belessi, J. Rung, N. Chiorazzi, J.C. Strefford, A.W. Langerak, S. Pospisilova, F. Davi, M. Hellstrom, H. Jernberg-Wiklund, P. Ghia, O. Soderberg, K. Stamatopoulos, M. Nilsson, R. Rosenquist, Functional loss of IkappaBepsilon leads to NF-kappaB deregulation in aggressive chronic lymphocytic leukemia, J Exp Med, 212 (2015) 833-843.

[12] L. Li, L. Li, M. Liu, Y. Li, Q. Sun, Novel immune-related prognostic model and nomogram for breast cancer based on ssGSEA, Front Genet, 13 (2022) 957675.

[13] J. Liu, J. Zhang, T-cell receptors provide potential prognostic signatures for breast cancer, Cell Biol Int, 45 (2021) 1220-1230.

[14] K. Pakos-Zebrucka, I. Koryga, K. Mnich, M. Ljujic, A. Samali, A.M. Gorman, The integrated stress response, EMBO Rep, 17 (2016) 1374-1395.

[15] S. Dabo, E.F. Meurs, dsRNA-dependent protein kinase PKR and its role in stress, signaling and HCV infection, Viruses, 4 (2012) 2598-2635.

[16] T.H. Kim, S.G. Cho, Kisspeptin inhibits cancer growth and metastasis via activation of EIF2AK2, Mol Med Rep, 16 (2017) 7585-7590.

[17] M.F. Griffin, M.R. Borrelli, J.T. Garcia, M. Januszyk, M. King, T. Lerbs, L. Cui, A.L. Moore, A.H. Shen, S. Mascharak, N.M. Diaz Deleon, S. Adem, W.L. Taylor, H.E. desJardins-Park, M. Gastou, R.A. Patel, B.A. Duoto, J. Sokol, Y. Wei, D. Foster, K. Chen, D.C. Wan, G.C. Gurtner, H.P. Lorenz, H.Y. Chang, G. Wernig, M.T. Longaker, JUN promotes hypertrophic skin scarring via CD36 in preclinical in vitro and in vivo models, Sci Transl Med, 13 (2021) eabb3312.

[18] M. Karin, Z. Liu, E. Zandi, AP-1 function and regulation, Curr Opin Cell Biol, 9 (1997) 240-246.

[19] A. Sundqvist, O. Voytyuk, M. Hamdi, H.E. Popeijus, C.B. van der Burgt, J. Janssen, J.W.M. Martens, A. Moustakas, C.H. Heldin, P. Ten Dijke, H. van Dam, JNK-Dependent cJun Phosphorylation Mitigates TGFbeta- and EGF-Induced Pre-Malignant Breast Cancer Cell Invasion by Suppressing AP-1-Mediated Transcriptional Responses, Cells, 8 (2019).

[20] L. Garcia-Martinez, A.M. Adams, H.L. Chan, Y. Nakata, N. Weich, S. Stransky, Z. Zhang, M. Alshalalfa, L. Sarria, B.A. Mahal, S.B. Kesmodel, T. Celia-Terrassa, Z. Liu, S. Minucci, D. Bilbao, S. Sidoli, R.E. Verdun, L. Morey, Endocrine resistance and breast cancer plasticity are controlled by CoREST, Nat Struct Mol Biol, 29 (2022) 1122-1135.

[21] M. Velasco-Velazquez, W. Xolalpa, R.G. Pestell, The potential to target CCL5/CCR5 in breast cancer, Expert Opin Ther Targets, 18 (2014) 1265-1275.

[22] A. Khalid, J. Wolfram, I. Ferrari, C. Mu, J. Mai, Z. Yang, Y. Zhao, M. Ferrari, X. Ma, H. Shen, Recent Advances in Discovering the Role of CCL5 in Metastatic Breast Cancer, Mini Rev Med Chem, 15 (2015) 1063-1072.

[23] M. Esmailbeig, A. Ghaderi, Interleukin-18: a regulator of cancer and autoimmune diseases, Eur Cytokine Netw, 28 (2017) 127-140.

[24] G. Tezcan, E.E. Garanina, M. Alsaadi, Z.E. Gilazieva, E.V. Martinova, M.I. Markelova, S.S. Arkhipova, S. Hamza, A. McIntyre, A.A. Rizvanov, S.F. Khaiboullina, Therapeutic Potential of Pharmacological Targeting NLRP3 Inflammasome Complex in Cancer, Front Immunol, 11 (2020) 607881.

[25] A. Rahman, L.S. Shashidhara, Analyzing the influence of IL18 in regulation of YAP1 in breast oncogenesis using cBioportal, Cancer Rep (Hoboken), 5 (2022) e1484.

[26] J.X. Shen, J. Liu, G.J. Zhang, Interleukin-33 in Malignancies: Friends or Foes?, Front Immunol, 9 (2018) 3051.

[27] H. Hu, J. Sun, C. Wang, X. Bu, X. Liu, Y. Mao, H. Wang, IL-33 facilitates endocrine resistance of breast cancer by inducing cancer stem cell properties, Biochem Biophys Res Commun, 485 (2017) 643-650.

[28] I.P. Jovanovic, N.N. Pejnovic, G.D. Radosavljevic, J.M. Pantic, M.Z. Milovanovic, N.N. Arsenijevic, M.L. Lukic, Interleukin-33/ST2 axis promotes breast cancer growth and metastases by facilitating intratumoral accumulation of immunosuppressive and innate lymphoid cells, Int J Cancer, 134 (2014) 1669-1682.

[29] O.S. Blomberg, L. Spagnuolo, H. Garner, L. Voorwerk, O.I. Isaeva, E. van Dyk, N. Bakker, M. Chalabi, C. Klaver, M. Duijst, K. Kersten, M. Bruggemann, D. Pastoors, C.S. Hau, K. Vrijland, E.A.M. Raeven, D. Kaldenbach, K. Kos, I.S. Afonina, P. Kaptein, L. Hoes, W. Theelen, P. Baas, E.E. Voest, R. Beyaert, D.S. Thommen, L.F.A. Wessels, K.E. de Visser, M. Kok, IL-5-producing CD4(+) T cells and eosinophils cooperate to enhance response to immune checkpoint blockade in breast cancer, Cancer Cell, 41 (2023) 106-123 e110.

[30] K.T. Tan, C.N. Yeh, Y.C. Chang, J.H. Cheng, W.L. Fang, Y.C. Yeh, Y.C. Wang, D.S. Hsu, C.E. Wu, J.I. Lai, P.M. Chang, M.H. Chen, M.L. Lu, S.J. Chen, Y. Chao, M. Hsiao, M.H. Chen, PRKDC: new biomarker and drug target for checkpoint blockade immunotherapy, J Immunother Cancer, 8 (2020).

[31] E. Cabanas Morafraile, J. Perez-Pena, J. Fuentes-Antras, A. Manzano, P. Perez-Segura, A. Pandiella, E.M. Galan-Moya, A. Ocana, Genomic Correlates of DNA Damage in Breast Cancer Subtypes, Cancers (Basel), 13 (2021).

[32] G. Sun, L. Yang, C. Dong, B. Ma, M. Shan, B. Ma, PRKDC regulates chemosensitivity and is a potential prognostic and predictive marker of response to adjuvant chemotherapy in breast cancer patients, Oncol Rep, 37 (2017) 3536-3542.

[33] Y. Zhang, W.K. Yang, G.M. Wen, H. Tang, C.A. Wu, Y.X. Wu, Z.L. Jing, M.S. Tang, G.L. Liu, D.Z. Li, Y.H. Li, Y.J. Deng, High expression of PRKDC promotes breast cancer cell growth via p38 MAPK signaling and is associated with poor survival, Mol Genet Genomic Med, 7 (2019) e908.

[34] J.L. Benci, L.R. Johnson, R. Choa, Y. Xu, J. Qiu, Z. Zhou, B. Xu, D. Ye, K.L. Nathanson, C.H. June, E.J. Wherry, N.R. Zhang, H. Ishwaran, M.D. Hellmann, J.D. Wolchok, T. Kambayashi, A.J. Minn, Opposing Functions of Interferon Coordinate Adaptive and Innate Immune Responses to Cancer Immune Checkpoint Blockade, Cell, 178 (2019) 933-948 e914.

[35] Z.H. Wu, Y. Tang, H. Yu, H.D. Li, The role of ferroptosis in breast cancer patients: a comprehensive analysis, Cell Death Discov, 7 (2021) 93.

[36] M. Oshi, M. Asaoka, Y. Tokumaru, L. Yan, R. Matsuyama, T. Ishikawa, I. Endo, K. Takabe, CD8 T Cell Score as a Prognostic Biomarker for Triple Negative Breast Cancer, Int J Mol Sci, 21 (2020).
